# A rapid volume of interest-based approach of radiomics analysis of breast MRI for tumor decoding and phenotyping of breast cancer

**DOI:** 10.1371/journal.pone.0234871

**Published:** 2020-06-26

**Authors:** Aydin Demircioglu, Johannes Grueneisen, Marc Ingenwerth, Oliver Hoffmann, Katja Pinker-Domenig, Elizabeth Morris, Johannes Haubold, Michael Forsting, Felix Nensa, Lale Umutlu

**Affiliations:** 1 Department of Diagnostic and Interventional Radiology and Neuroradiology, University Hospital Essen, University of Duisburg-Essen, Essen, Germany; 2 Department of Pathology, University Hospital Essen, University of Duisburg-Essen, Essen, Germany; 3 Department of Gynaecology and Obstetrics, University Hospital Essen, University of Duisburg-Essen, Essen, Germany; 4 Breast Imaging Service, Memorial Sloan Kettering Cancer Center, New York, NY, United States of America; Medical University of Vienna, AUSTRIA

## Abstract

**Background:**

Recently, radiomics has emerged as a non-invasive, imaging-based tissue characterization method in multiple cancer types. One limitation for robust and reproducible analysis lies in the inter-reader variability of the tumor annotations, which can potentially cause differences in the extracted feature sets and results. In this study, the diagnostic potential of a rapid and clinically feasible VOI (Volume of Interest)-based approach to radiomics is investigated to assess MR-derived parameters for predicting molecular subtype, hormonal receptor status, Ki67- and HER2-Expression, metastasis of lymph nodes and lymph vessel involvement as well as grading in patients with breast cancer.

**Methods:**

A total of 98 treatment-naïve patients (mean 59.7 years, range 28.0–89.4) with BI-RADS 5 and 6 lesions who underwent a dedicated breast MRI prior to therapy were retrospectively included in this study. The imaging protocol comprised dynamic contrast-enhanced T1-weighted imaging and T2-weighted imaging. Tumor annotations were obtained by drawing VOIs around the primary tumor lesions followed by thresholding. From each segmentation, 13.118 quantitative imaging features were extracted and analyzed with machine learning methods. Validation was performed by 5-fold cross-validation with 25 repeats.

**Results:**

Predictions for molecular subtypes obtained AUCs of 0.75 (HER2-enriched), 0.73 (triple-negative), 0.65 (luminal A) and 0.69 (luminal B). Differentiating subtypes from one another was highest for HER2-enriched vs triple-negative (AUC 0.97), followed by luminal B vs triple-negative (0.86). Receptor status predictions for Estrogen Receptor (ER), Progesterone Receptor (PR) and Hormone receptor positivity yielded AUCs of 0.67, 0.69 and 0.69, while Ki67 and HER2 Expressions achieved 0.81 and 0.62. Involvement of the lymph vessels could be predicted with an AUC of 0.8, while lymph node metastasis yielded an AUC of 0.71. Models for grading performed similar with an AUC of 0.71 for Elston-Ellis grading and 0.74 for the histological grading.

**Conclusion:**

Our preliminary results of a rapid approach to VOI-based tumor-annotations for radiomics provides comparable results to current publications with the perks of clinical suitability, enabling a comprehensive non-invasive platform for breast tumor decoding and phenotyping.

## Background

Breast cancer is one of the most common causes of cancer-related death in women worldwide. The prognosis depends on the time of first diagnosis and treatment, which in turn strongly depends on the molecular and hormonal features of the tumor. In clinical routine, these features are assessed by invasive tissue sampling and histopathological analysis. Over the past few years, ‘radiomics’ or ‘radiogenomics’ has demonstrated its potential for non-invasive, imaging-based tissue characterization in multiple cancer types [1–4]. As a rapidly emerging field, radiomics is based on the extraction of numerous quantitative features from medical images to assess relationships between features and the underlying pathophysiology using machine learning methods for analysis [5]. While radiomics-based analysis for prediction of the molecular status has been shown highly successful for brain cancers and prostate cancer [6–8], results from previously published studies on breast cancer show promising, yet divergent results [9,10]. One limitation for robust and reproducible radiomics analysis lies in the impact of widely-divergent inter-reader variability in annotating the tumors [11]. To date most radiomics studies require an either fully manual or semi-automatic tumor segmentation by a radiologist to enable the feature extraction process [10,12–16], resulting in potentially strongly divergent differences of the tumor annotations. These differences have been shown to cause significant variations in the feature sets extracted from the annotated tumor images and the consequential results thereof.

Therefore, the aim of this study was to investigate whether the utilization of a rapid and reproducible approach of VOI-based semi-automatic radiomics can facilitate the prediction of the molecular subtype, hormonal receptor status, Ki67- and HER2-Expression, involvement of lymph nodes and lymph vessel involvement as well as grading in patients with breast cancer.

## Materials and methods

### Patients

Ethical approval for this retrospective study was granted by the local ethics committee. Informed consent was waived. All analyses were performed based on anonymized data. After a prospective database research a total of 114 patients with histopathological confirmation of breast cancer (BIRADS 5 or 6) between August 2014 and March 2018, who fulfilled the following criteria were included in this study: (1) dedicated breast MRI prior treatment, (2) breast MRI performed exclusively on one dedicated 1.5 Tesla MR scanner to reduce potential influence of varying field strengths or imaging parameters on texture analysis, (3) lesions >/ = 1cm on MRI to decrease potential partial volume effects on radiomic analysis [17], (4) 18 years or older, (5) neither pregnant nor breastfeeding. 16 of these 114 patients with incomplete MR sequences were excluded from this study, resulting in a total of 98 patients who were included in the analysis.

### Data acquisition

Breast MR scans were performed on a 1.5 Tesla MR system (Aera, Siemens Healthcare, Erlangen) in prone position utilizing a dedicated 16-channel breast radiofrequency (RF) coil (Siemens Healthcare, Erlangen).

The study protocol comprised the following sequences:

- A transversal 2-dimensional turbo-spin echo sequence (0.7 x 0.7 mm voxel size, 2.0 mm slice thickness, gap 0 mm, TR 6100 ms, TE 52 ms, FOV 340 x 340 mm^2^, flip angle 150 degrees, matrix size 256x256, 88 slices)
- Six repetitions of a non-fatsaturated transversal 3-dimensional fast low-angle shot (FLASH) sequence for dynamic imaging (0.3 x 0.3 mm voxel size, 2.0 mm slice thickness, gap 0 mm, TR 8.7 ms, TE 4.76 ms, FOV 400 x 400 mm^2^, flip angle 25 degrees, matrix size 256x256, 88 slices). A dose of 0.1 mmol/kg bodyweight gadobutrol (Bayer Healthcare, Berlin, Germany) was injected intravenously after the first FLASH sequence with a flow of 2 mL/s using an automated injector (Spectris Solaris, MR Injection System; Medrad, Pittsburg, PA).
- Subtraction imaging was acquired automatically after completion of the dynamic series.

### Standard of reference

Histopathological analysis served as the reference standard and was based on either core needle biopsy (n = 79) or surgical resection (n = 19). Histological and molecular subtype classification was determined according to the 2013 St. Gallen guidelines [18]. IHC (ImmunoHistoChemistry) status analysis comprised estrogen receptor, progesterone receptor and HER2 status. Positivity for ER and PR was defined as the presence of 1% or more of positively stained nuclei. Molecular subtypes were classified accordingly: (1) luminal A for hormone receptor positive and HER2 negative, (2) luminal B for hormone receptor positive and HER2 positive, (3) triple-negative for hormone receptor and HER2 negative and (4) HER2-enriched for hormone receptor negative and HER2 positive [19–21]. Histological grading was determined in accordance with Elston-Ellis [22]. Elston-Ellis gradings 5, 6 and 7 were classified into a low-grade group, gradings 8 and 9 into a high-grade group. Ki67 was considered to be low, if the value was below 14% and high if otherwise [18]. A list of all outcome variables is displayed in Table 1.

**Table 1:** Display of the distribution of the outcome variables. N denotes the sample size used for each outcome, while Positive and Negative denote the balance of the data.

Outcome Variable	N	Positive	Negative
*Molecular breast cancer subtypes*			
Luminal A	95	58 (61%)	37 (39%)
Luminal B	95	16 (17%)	79 (83%)
HER2-enriched	95	6 (6%)	89 (94%)
Triple-Negative	95	15 (16%)	80 (84%)
*Differentiation of Molecular breast cancer subtypes*			
Luminal A vs Luminal B	74	58 (78%)	16 (22%)
Luminal A vs HER2-enriched	64	58 (91%)	6 (9%)
Luminal A vs Triple- Negative	73	58 (79%)	15 (21%)
Luminal B vs HER2-enriched	22	16 (73%)	6 (27%)
Luminal B vs Triple- Negative	31	16 (52%)	15 (48%)
HER2-enriched vs Triple-Negative	21	6 (29%)	15 (71%)
*Receptor status*			
Estrogen Receptor (ER)	95	73 (77%)	22 (23%)
Progesterone Receptor (PR)	95	66 (69%)	29 (31%)
Hormone receptor positivity	95	74 (78%)	21 (22%)
*Ki67- and HER2-Expression*			
Ki67	80	20 (25%)	60 (75%)
Human epidermal growth factor receptor 2 (HER2)	95	22 (23%)	73 (77%)
*Involvement of the lymphatic system*			
Lymph Vessel Involvement	51	8 (16%)	43 (84%)
Lymph Node Metastasis	84	34 (40%)	50 (60%)
*Grading*			
Elston-Ellis Grading (EE)	57	44 (77%)	13 (23%)
Histological Grading	90	18 (20%)	72 (80%)

### Radiomics and statistical analysis

**Fig 1:**
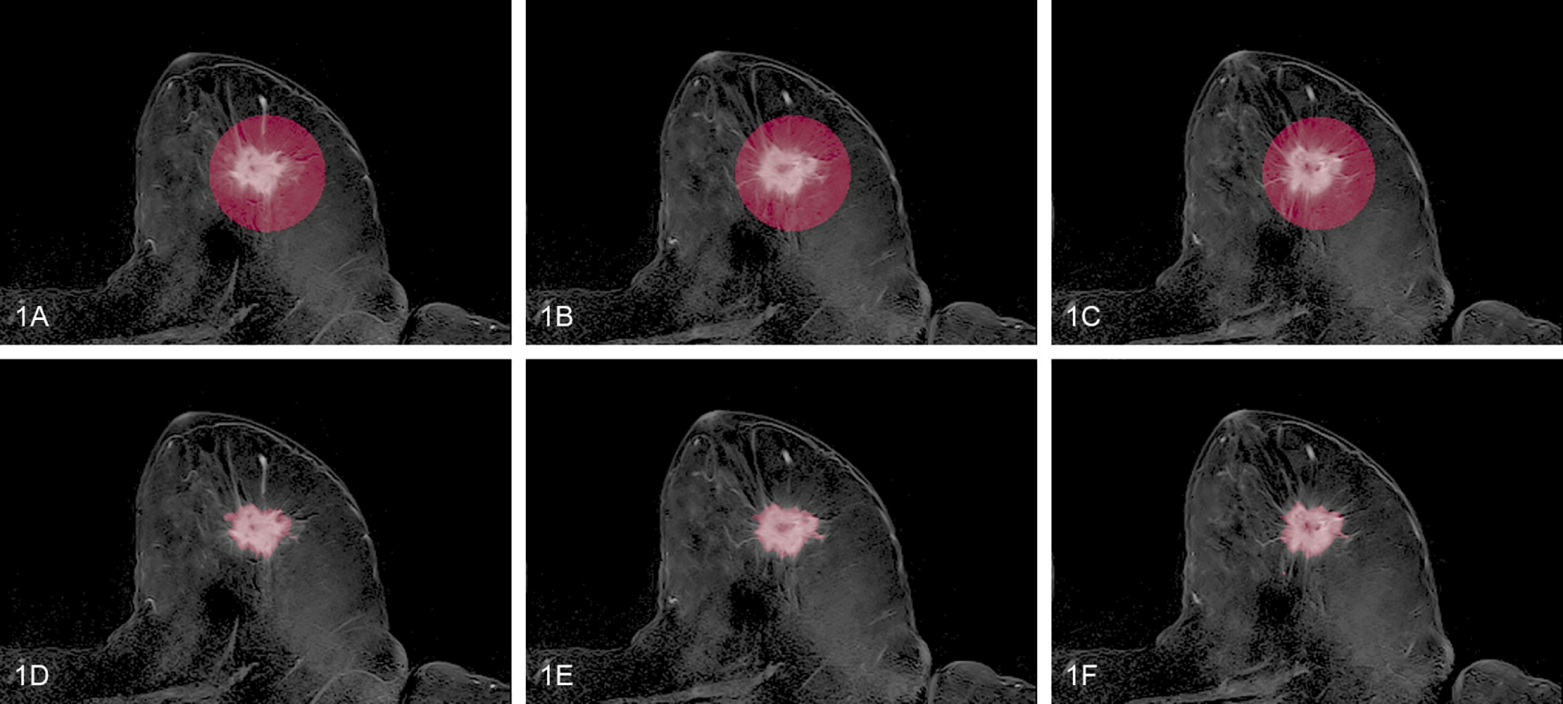
Imaging example of the VOI-encirclement of a tumor lesion. Fig 1A–1C display three consecutive axial slices of a tumor lesion in the second subtraction series annotated via VOI-encirclement. Fig 1D–1F show the corresponding semi-automatic fine tumor segmentation obtained by thresholding.

All MRI datasets were imported into the open source image processing software 3D-Slicer [23]. To ensure best conspicuity of the cancerous lesions the second subtraction series of the dynamic set was utilized for radiomic analysis. Tumor volumes were encircled utilizing a VOI (volume of interest) by an experienced breast radiologist with 15 years of experience (Fig 1A). A simple threshold of using 30% of the highest intensity inside the volume, followed with a morphological closing was used to obtain an automatic fine segmentation of the tumor mass (Fig 1B). Since this method may generate several segments, only the largest was retained. All segmentations were then reviewed by the above mentioned experienced breast radiologist and exported as DICOM-SEG. (http://dicom.nema.org/medical/dicom/current/output/chtml/part03/sect_A.51.html).

Image preprocessing was performed in accordance with previous publications [1,5,24], e.g. a gradient filter was used to make the T1-weighted DCE-MRI series and the T2-weighted series more comparable. Overall, 13.118 generic features comprising shape, first order and higher order features were generated (a detailed list is displayed in S1 File). Five different feature selection methods (randomized logistic regression, chi-square, f-score, t-score, mutual information) were used to reduce the set of features. For machine learning analysis, three different classifiers (Naive Bayes, random forests and logistic regression) were employed. Classification performance was measured using a Receiver Operating Characteristic (ROC) analysis and reported as Area under the Curve (AUC) with a 95% confidence interval. A two-sided DeLong test was employed to compare if the AUC of ROC curves is different to that of a constant predictor. More details are listed in S3 File.

All analyses were performed using Python 3.6. Pyradiomics 2.0.1 [24] was used for the extraction of the radiomics features. Implementations of the feature selection methods and classifiers were taken from the scikit-learn 0.21.0 package.

### Validation

For internal validation, a stratified 5-fold cross-validation (CV) was applied, selecting 80% of the samples randomly in each round for training, with the remaining 20% being reserved exclusively for testing. Feature selection was applied only to the training folds. The CV was repeated 25 times to get a more accurate estimation of the final performance. As some of the outcome variables showed imbalanced positive and negative classes (Table 1), stratified sampling was used.

The prediction capacity of all models was measured by averaging over the corresponding cross-validation test folds. Subsequently, AUC as well as sensitivity and specificity were computed.

## Results

A total of 98 patients (mean age 59.7, range 28.0–89.4) were included in the study. Mean lesion size was 4.9 +/- 2.9 cm (range: 1.2–16.1 cm).

Due to low occurrence, patients with an Elston-Ellis grading below 5 (n = 3) or with a histopathological grading of 1 (n = 2) were removed from the corresponding subanalyses. The sample sizes of all subanalyses ranged from 21 to 95. The distributions of the outcome variables are listed in Table 1.

**Fig 2:**
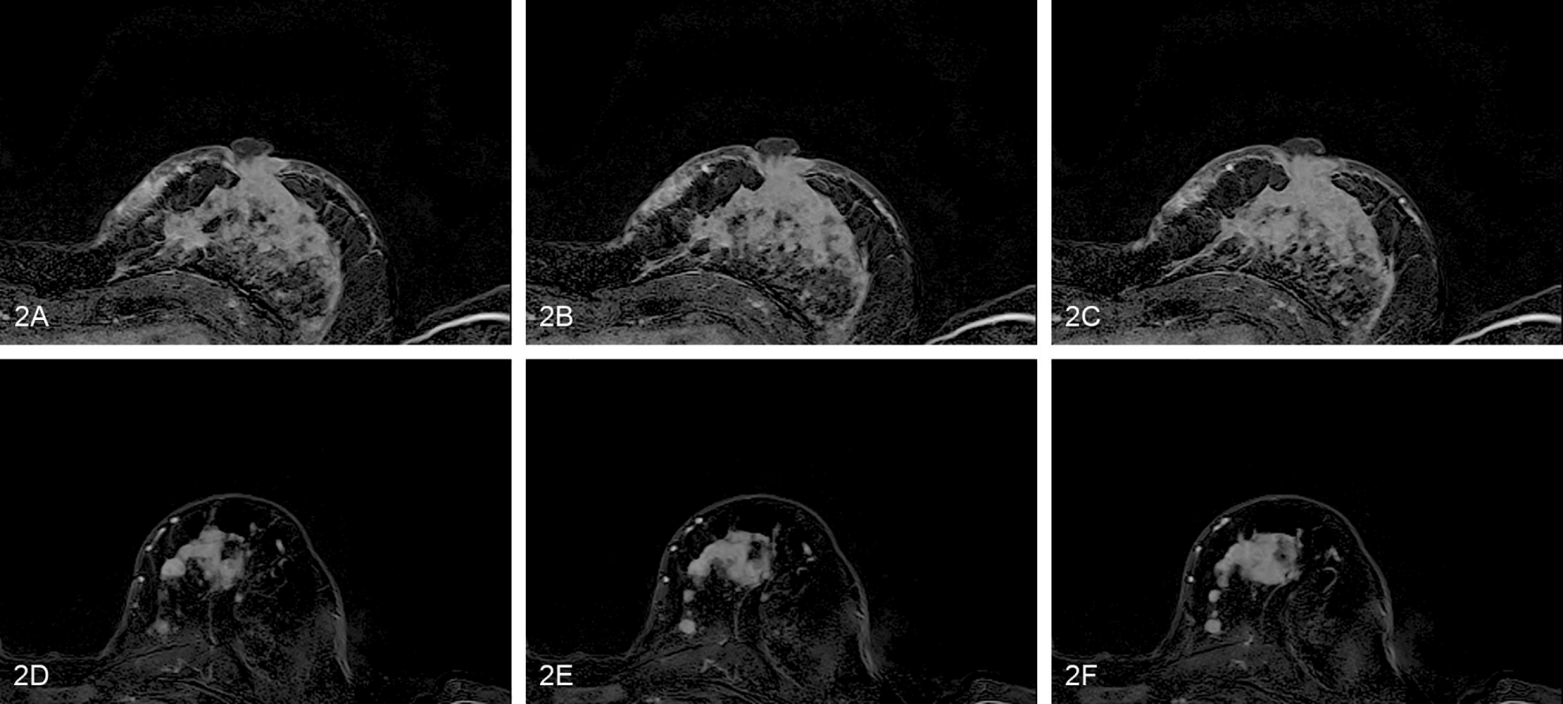
Example of two patients with low and high prediction scores using the model trained to predict low vs high Ki67. (A-C) Three consecutive axial slices of a tumor lesion in the second subtraction series for the patient with the highest prediction score. Histological reference revealed a Ki67 count of 80. (D-F) The corresponding axial slices for the patient with the lowest prediction score. Histological reference revealed a Ki67 count of 10.

Trained models produced prediction scores that were used for the ROC analysis (Fig 2 and violin plots in S4 File). Predictions showed varying results, ranging from AUC 0.62 to 0.97. Detailed results of classification accuracies are displayed in Table 2 and visualized in ROC plots in S5 File.

**Table 2:** Detailed results of the classification accuracies. Results for all classifiers. P-Values were computed using a DeLong test to compare ROC curves.

Outcome	Classifier	Feature Selection Method	Number of Features	AUC [95% CI], p-Value	Sensitivity	Specificity	Accuracy
*Molecular breast cancer subtypes*							
Luminal A	Random forest	Mutual Information	32	0.65 [0.54, 0.77], p = 0.009	0.53	0.78	0.62
Luminal B	Random forest	t-Score	32	0.69 [0.55, 0.84], p = 0.008	0.56	0.76	0.72
HER2-enriched	Logistic Regression	Randomized Logistic Regression	2	0.75 [0.58, 0.91], p = 0.003	0.67	0.82	0.80
Triple- Negative	Random forest	Chi Square	32	0.73 [0.58, 0.87], p = 0.002	0.8	0.6	0.59
*Differentiation of Molecular breast cancer subtypes*							
Luminal A vs Luminal B	Random forest	Randomized Logistic Regression	4	0.64 [0.47, 0.81], p = 0.115	0.78	0.56	0.72
Luminal A vs HER2-enriched	Random forest	Mutual Information	16	0.79 [0.58, 0.99], p = 0.003	0.79	0.83	0.78
Luminal A vs Triple- Negative	Random forest	t-Score	8	0.74 [0.61, 0.87], p < 0.001	0.66	0.8	0.66
Luminal B vs HER2-enriched	Logistic Regression	F-Score	2	0.78 [0.59, 0.97], p = 0.003	0.69	0.83	0.68
Luminal B vs Triple- Negative	Naive Bayes	Randomized Logistic Regression	1	0.86 [0.71, 1.0], p < 0.001	0.88	0.87	0.84
HER2-enriched vs Triple Negative	Naive Bayes	Mutual Information	2	0.97 [0.89, 1.0], p < 0.001	0.83	1.0	0.90
*Receptor status*							
Estrogen Receptor (ER)	Naive Bayes	Mutual Information	32	0.67 [0.53, 0.8], p = 0.014	0.68	0.68	0.63
Progesterone Receptor (PR)	Naive Bayes	t-Score	32	0.69 [0.57, 0.8], p = 0.002	0.71	0.62	0.69
Hormone receptor positivity	Logistic Regression	Chi Square	1	0.69 [0.57, 0.81], p = 0.002	0.65	0.71	0.61
*Ki67- and HER2-Expression*							
Ki67	Logistic Regression	Randomized Logistic Regression	8	0.81 [0.7, 0.92], p < 0.001	0.75	0.68	0.84
Human epidermal growth factor receptor 2 (HER2)	Random forest	Randomized Logistic Regression	16	0.62 [0.48, 0.75], p = 0.079	0.64	0.58	0.51
*Involvement of the lymphatic system*							
Lymph Vessel Involvement	Random forest	Mutual Information	8	0.8 [0.65, 0.95], p < 0.001	0.88	0.67	0.68
Lymph Node Metastasis	Logistic Regression	Mutual Information	1	0.71 [0.6, 0.83], p < 0.001	0.71	0.74	0.71
*Grading*							
Elston-Ellis Grading (EE)	Logistic Regression	Chi Square	32	0.71 [0.55, 0.88], p = 0.009	0.64	0.85	0.67
Histological Grading	Naive Bayes	Randomized Logistic Regression	16	0.74 [0.62, 0.87], p < 0.001	0.72	0.72	0.62

### Molecular breast cancer subtypes

Highest accuracies for correct classification were achieved for the differentiation of HER2-enriched from triple-negative with an AUC of 0.97, followed by luminal B from triple-negative (AUC 0.86) and luminal A and B from HER2-enriched (AUC 0.79 and 0.78 respectively). Classification of the individual molecular subtype against all other subtypes showed moderate to low accuracies, with best accuracies achieved for prediction of HER2-enriched (AUC 0.75) and lowest for luminal A (AUC 0.65).

### Receptor status

Classification of the hormonal receptor status was highest for the pairwise discrimination of the overall hormone receptor positivity and Progesterone receptor positivity with AUCs of 0.69. Comparable, yet slightly lower AUCs were achieved for Estrogen receptor positivity with an AUC of 0.67.

### Ki67- and HER2-expression

Prediction of Ki67 Expression was achieved with an AUC of 0.81. Classification of HER2-Expression was rather low with an AUC of 0.62.

### Involvement of the lymphatic system

Prediction of lymph vessel involvement achieved high accuracies (AUC 0.80), while classification of involvement of lymph nodes was moderate with an AUC of 0.72.

### Grading

Both gradings (histological grading and Elston-Ellis grading) showed comparably moderate performance, with AUC 0.71 for the prediction of the Elston-Elliis grading and 0.75 for histological grading.

### Features and feature selection methods

Regarding the number of features, no general trend could be observed (a detailed list of the selected features is displayed in S2 File while a correlation matrix of all extracted features with the outcomes is visualized in S6 File). The number of selected features of the best models differed for each outcome variable, e.g. selecting 1 feature yielded the best model for lymph node metastasis, while the model using 32 features showed the best performance for prediction of the Elston-Ellis grading. Similarly, the most often selected features differed: Considering only those 114 features that have been selected more than 50% in all training repeats across all sub-analyses, most often features from the DCE-MRI sequence were selected (70%, N = 80) with lowest features derived from T2 imaging (11%, N = 12). Considering the preprocessing, wavelet features were most often used (41%, N = 47), followed by local binary pattern features (24%, N = 27). No shape features were selected by any of the feature selection methods. While most often first order features (36%, N = 41) and GLSZM as well as GLCM features (22%, N = 25 and 21%, N = 24 resp.) were used, no clear pattern can be seen in the features themselves. Here, the most often selected feature was LargeAreaHighGrayLevelEmphasis (8%, N = 9), while variants of the emphasis feature was most often used (32%, N = 36).

No clear pattern was observed regarding the feature selection method and the classifier. Models using randomized logistic regression (N = 6) were equally successful in producing best performing models as mutual information (N = 6). Similarly, no superiority of any of the classifiers was seen: Though random forests were used slightly more often than logistic regression (N = 8 and N = 6), both were not outperforming Naive Bayes (N = 5).

## Discussion

Inter- and intratumoral heterogeneity is one of the hallmarks of breast cancer [25] having induced refinements of the previously mainly pathology-driven classification to molecular classifications and subtyping [26]. Each of these subtypes is associated to different risk factors for incidence, response to treatment, risk of disease progression, and preferential organ sites of metastases. Luminal tumors are positive for estrogen and progesterone receptors, and the majority respond well to hormonal interventions, whereas HER2^+^ tumors have amplification and overexpression of the *ERBB2* oncogene and can be effectively controlled with a diverse array of anti-HER2 therapies. Basal-like tumors in general lack hormone receptors and HER2; thus, the majority of these tumors are also called triple-negative breast cancer (TNBC). While the “personalization” of treatment, by means of adaptation in accordance with molecular subtyping, has significantly changed and improved breast cancer treatment, invasive tissue sampling may be restricted in its assessment of whole-tumor heterogeneity, as particularly in patients undergoing neoadjuvant chemotherapy only a minor part of the tumor is histopathologically analyzed prior to treatment.

Over the past few years, Radiomics has been demonstrated to facilitate a promising platform for non-invasive whole-tumor tissue characterization for a number of cancer types [6,7,27]. An increasing number of research studies have found radiomic signatures to be predictive markers of underlying gene-expression patterns, therapy response, relapse, patient survival and other clinical and histopathological outcomes, building a bridge between imaging and genomics, also known as radiogenomics [4,6,28]. In one of the largest studies on molecular subtyping, Saha et al. [9] extracted 529 features from DCE-MRI imaging of 922 patients and predicted luminal A, triple-negative, ER and PR status with AUCs of 0.70, 0.66, 0.66 and 0.62, respectively. These results coincide well with our study results, with the biggest difference lying in the prediction of triple-negative cancers (AUC 0.66 vs 0.73). Similarly, in a smaller study, Guo et al. [10] predict Stage I vs III, ER, PR, HER2 as well as lymph node metastases (LNM) from radiological and genetic data. The AUCs of the radiomics only models were 0.87, 0.79, 0.69, 0.65 and 0.69 for Stage, ER, PR, HER2 and LNM respectively. Again, these numbers lie within the 95% CI of our models, differing strongest in prediction of ER (AUC 0.79 vs 0.67). Our results in differentiation of molecular subtypes also go in line with a previous publication by Leithner et al. [29], revealing comparable results for the differentiation of luminal A from HER2-enriched, luminal B from triple-negative and superior results for distinguishing HER2-enriched from triple-negative cancers.

Similar results have also been obtained using computer-assisted diagnostics (CAD), where instead of a broad set of automatically generated features only a few manual ones are extracted and analyzed via statistical or machine learning models. Baltzer et al. [30] demonstrated that volume enhancement characteristics are significantly associated with LNM, ER, PR and HER2. Dietzel et al. [31] used a neural network to predict lymph node status and obtained an AUC of 0.74, which is comparable to our result.

Nevertheless, our study differs from a number of previous trials in three important factors: Firstly, we favored a rather simple and robust imaging protocol for analysis, comprising dynamic T1-weighted imaging and T2-weighted imaging. While DWI is known to be susceptible to artifacts and suffer from an overall lack of standardization, it is considered as an important source of functional data and has been shown to improve specificity [32]. Nonetheless DWI was not used in this study as it was not part of our standard imaging protocol. As it was not consistently available for all patients, it was excluded from our analysis. Secondly, our approach to non-invasive tissue characterization was composed broader than most previous studies in comprising the molecular subtype as well as parameters like Ki67, grading and involvement of lymph tissue. While the prediction of grading and involved lymph nodes may be considered rather moderate with AUCs of 0.71 and 0.75, respectively, prediction of Ki67 achieved clinically relevant and high AUCs of 0.81. Ki67 expression and Ki67 index have been shown to play considerable roles as predictive factors for disease free survival, overall survival and may be used for treatment and follow-up assessment [33]. The third and most distinctive difference is our approach towards tumor annotation. Most previous radiomics studies were based on labor-intensive manual or semi-automatic full-tumor annotations performed by the radiologists, resulting in potential inter-reader variabilities depending on each radiologists´ appreciation of the tumor shape and size as well as expertise. Our approach was simplified in restricting the radiologists´ influence to recognition and a VOI-based encirclement of the tumor and a subsequent sub-automatic segmentation by the machine learning algorithm, using a simple threshold of 30% of the highest intensity inside the volume to obtain an automatic fine segmentation of the tumor mass. A similar segmentation approach was adopted recently by Dietzel et al. [34]. By thresholding voxels in the initial enhancement in the dynamics having more than 30% difference they identify the active tumor tissue and extract vascularization patterns.

This simplified approach endorses two important factors: first, the time for tumor annotation is significantly reduced from a dedicated whole-tumor manual / semi-automatic annotation to a simple VOI demarcation as commonly used in hybrid imaging for assessment of tracer uptake, making this approach feasible in clinical routine. Secondly, these VOI and intensity-based segmentations reduce potential inter-reader variabilities based on manual / semi-automatic segmentation by radiologist. This simplified approach to radiomics goes in line with the recent recognition of the potential impact of inter-reader variability in radiomics. Saha et al. [11] recently published an overview of algorithmic features and the potential impact of inter-reader variability in annotating tumors. They conclude that breast MRI radiomics features widely vary in terms of their stability, ranging from an average inter-reader stability for all features of 0.85 to as low as 0.63 for tumor-based features.

Compared to previous publications [35,36] our study results do not facilitate clear recommendations regarding the feature selection method or the applied classifiers. This underlines that in high dimensional spaces no method generally outperforms others. This also holds true for the feature selection, where similarly no pattern could be clearly detected. Overall, dynamic T1-weighted imaging seems to facilitate the extraction of more relevant features than T2 weighted imaging, which goes in line with previous publications on this matter [32,37].

While the present study offers several strong points, including a rather broad scope and simplified approach to tumor-annotation, some limitations apply: The small sample size might have introduced a bias in form of overfitting and impeded the splitting of the data into strict train and test sets. This stems from the fact that the lesions of many patients were clip-marked at the time of examination and thus were excluded from the study. Furthermore, a total of 24 patients with BIRADS 6 were included. As a biopsy was performed in these patients prior to imaging, an effect on the radiomics analysis cannot be ruled out. A meaningful subgroup analysis would require a higher sample size and was thus not carried out. Another bias may be caused by the fact, that all examinations in this study were performed with a scanner from a single provider. Finally, due to the “proof-of-concept” nature of this study, another restriction may be elicited by the lack of an external validation data set, as the primary study goal was to establish a simplified approach to radiomics. Hence, all of these weak points should be addressed in future trials, comprising larger patient cohorts of multi-center, multi-vendor studies.

## Conclusions

Overall, our preliminary study results demonstrate the usability of a simplified and rapid approach to tumor for MRI-based tumor decoding and phenotyping of breast cancer.

## Supporting information

S1 File List of extracted features. This file contains a list of the 13,118 extracted features as well as the applied preprocessing methods.
(DOCX)

S2 File List of selected features. This file contains a list of features that were selected by the best performing methods.
(DOCX)

S3 File Analysis pipeline. This file contains a detailed description of the performed analysis.
(DOCX)

S4 File Violin plots. This file contains all violin plots for the prediction scores.
(DOCX)

S5 File ROC curves. This file contains all ROC curves.
(DOCX)

S6 File Correlations of extracted features with outcomes. This file contains a plot depicting the correlation of all extracted features with the outcomes.
(DOCX)
