# Supplementary material for: A rapid volume of interest-based approach of radiomics analysis of breast MRI for tumor decoding and phenotyping of breast cancer
Source: PLoS One. 2020 Jun 26;15(6):e0234871. doi: 10.1371/journal.pone.0234871 (PMC7319601; doi:10.1371/journal.pone.0234871)
Supplement: S1 File — This file contains a list of the 13,118 extracted features as well as the applied preprocessing methods. (DOCX) [file pone.0234871.s001.docx]

**Supplementary File 1**

**List of extracted features**

All scans were first preprocessed with different methods allowing the analysis to focus on different aspects and then, based on previous experience, quantized with a bin count of 24. From each segment 13,118 generic features comprising of shape, first order and higher order features were generated.

The following 7 series were used:

- T1 native

- T1 DCE series 1

- T1 DCE series 2

- T1 DCE series 3

- T1 DCE series 4

- T1 DCE series 5

- T2 weighted

The following 20 preprocessing filters were applied to each of the series:

- exponential

- gradient

- lbp-2D

- lbp-3D-k

- lbp-3D-m1

- lbp-3D-m2

- log-sigma-0-1-mm-3D

- log-sigma-1-0-mm-3D

- logarithm

- original

- square

- squareroot

- wavelet-HHH

- wavelet-HHL

- wavelet-HLH

- wavelet-HLL

- wavelet-LHH

- wavelet-LHL

- wavelet-LLH

- wavelet-LLL

From each series 1874 features were extracted.

Features were extracted from the following classes:

- firstorder

- glcm

- gldm

- glrlm

- glszm

- ngtdm

- shape

From each preprocessed series the following 93 features were extracted:

- firstorder: 10Percentile

- firstorder: 90Percentile

- firstorder: Energy

- firstorder: Entropy

- firstorder: InterquartileRange

- firstorder: Kurtosis

- firstorder: Maximum

- firstorder: Mean

- firstorder: MeanAbsoluteDeviation

- firstorder: Median

- firstorder: Minimum

- firstorder: Range

- firstorder: RobustMeanAbsoluteDeviation

- firstorder: RootMeanSquared

- firstorder: Skewness

- firstorder: TotalEnergy

- firstorder: Uniformity

- firstorder: Variance

- glcm: Autocorrelation

- glcm: ClusterProminence

- glcm: ClusterShade

- glcm: ClusterTendency

- glcm: Contrast

- glcm: Correlation

- glcm: DifferenceAverage

- glcm: DifferenceEntropy

- glcm: DifferenceVariance

- glcm: Id

- glcm: Idm

- glcm: Idmn

- glcm: Idn

- glcm: Imc1

- glcm: Imc2

- glcm: InverseVariance

- glcm: JointAverage

- glcm: JointEnergy

- glcm: JointEntropy

- glcm: MCC

- glcm: MaximumProbability

- glcm: SumAverage

- glcm: SumEntropy

- glcm: SumSquares

- gldm: DependenceEntropy

- gldm: DependenceNonUniformity

- gldm: DependenceNonUniformityNormalized

- gldm: DependenceVariance

- gldm: GrayLevelNonUniformity

- gldm: GrayLevelVariance

- gldm: HighGrayLevelEmphasis

- gldm: LargeDependenceEmphasis

- gldm: LargeDependenceHighGrayLevelEmphasis

- gldm: LargeDependenceLowGrayLevelEmphasis

- gldm: LowGrayLevelEmphasis

- gldm: SmallDependenceEmphasis

- gldm: SmallDependenceHighGrayLevelEmphasis

- gldm: SmallDependenceLowGrayLevelEmphasis

- glrlm: GrayLevelNonUniformity

- glrlm: GrayLevelNonUniformityNormalized

- glrlm: GrayLevelVariance

- glrlm: HighGrayLevelRunEmphasis

- glrlm: LongRunEmphasis

- glrlm: LongRunHighGrayLevelEmphasis

- glrlm: LongRunLowGrayLevelEmphasis

- glrlm: LowGrayLevelRunEmphasis

- glrlm: RunEntropy

- glrlm: RunLengthNonUniformity

- glrlm: RunLengthNonUniformityNormalized

- glrlm: RunPercentage

- glrlm: RunVariance

- glrlm: ShortRunEmphasis

- glrlm: ShortRunHighGrayLevelEmphasis

- glrlm: ShortRunLowGrayLevelEmphasis

- glszm: GrayLevelNonUniformity

- glszm: GrayLevelNonUniformityNormalized

- glszm: GrayLevelVariance

- glszm: HighGrayLevelZoneEmphasis

- glszm: LargeAreaEmphasis

- glszm: LargeAreaHighGrayLevelEmphasis

- glszm: LargeAreaLowGrayLevelEmphasis

- glszm: LowGrayLevelZoneEmphasis

- glszm: SizeZoneNonUniformity

- glszm: SizeZoneNonUniformityNormalized

- glszm: SmallAreaEmphasis

- glszm: SmallAreaHighGrayLevelEmphasis

- glszm: SmallAreaLowGrayLevelEmphasis

- glszm: ZoneEntropy

- glszm: ZonePercentage

- glszm: ZoneVariance

- ngtdm: Busyness

- ngtdm: Coarseness

- ngtdm: Complexity

- ngtdm: Contrast

- ngtdm: Strength

For the unprocessed series (called “original’) additionally the following 14 features were extracted.

- Elongation

- Flatness

- LeastAxisLength

- MajorAxisLength

- Maximum2DDiameterColumn

- Maximum2DDiameterRow

- Maximum2DDiameterSlice

- Maximum3DDiameter

- MeshVolume

- MinorAxisLength

- Sphericity

- SurfaceArea

- SurfaceVolumeRatio

- VoxelVolume
