# Supplementary material for: A rapid volume of interest-based approach of radiomics analysis of breast MRI for tumor decoding and phenotyping of breast cancer
Source: PLoS One. 2020 Jun 26;15(6):e0234871. doi: 10.1371/journal.pone.0234871 (PMC7319601; doi:10.1371/journal.pone.0234871)
Supplement: S2 File — This file contains a list of features that were selected by the best performing methods. (DOCX) [file pone.0234871.s002.docx]

**Supplementary File 2**

**List of selected features**

114 Features were selected overall.

Series

22 (19.0%) Features from the T1 native series were selected.

12 (11.0%) Features from the T2 series were selected.

80 (70.0%) Features from the DCE-MRI series were selected.

Preprocessing

Features from wavelet-LLL were selected 3 (3.0%)

Features from wavelet-HLH were selected 2 (2.0%)

Features from wavelet-HLL were selected 7 (6.0%)

Features from wavelet-HHH were selected 2 (2.0%)

Features from original were selected 7 (6.0%)

Features from lbp-3D-m2 were selected 16 (14.0%)

Features from gradient were selected 11 (10.0%)

Features from lbp-3D-m1 were selected 4 (4.0%)

Features from square were selected 1 (1.0%)

Features from log-sigma-0-1-mm-3D were selected 2 (2.0%)

Features from wavelet-LLH were selected 1 (1.0%)

Features from lbp-2D were selected 7 (6.0%)

Features from exponential were selected 1 (1.0%)

Features from squareroot were selected 9 (8.0%)

Features from wavelet-LHL were selected 25 (22.0%)

Features from wavelet-HHL were selected 1 (1.0%)

Features from wavelet-LHH were selected 6 (5.0%)

Features from logarithm were selected 9 (8.0%)

Feature Order

Features from glrlm were selected 7 (6.0%)

Features from gldm were selected 15 (13.0%)

Features from glszm were selected 25 (22.0%)

Features from glcm were selected 24 (21.0%)

Features from ngtdm were selected 2 (2.0%)

Features from firstorder were selected 41 (36.0%)

Top Features

Features from 10Percentile were selected 6 often (5.0%)

Features from LargeAreaHighGrayLevelEmphasis were selected 9 often (8.0%)

Features from Maximum were selected 6 often (5.0%)

Features from Median were selected 7 often (6.0%)

Features from Range were selected 6 often (5.0%)

Emphasis features were selected 36 often (32.0%)
