# Supplementary material for: A rapid volume of interest-based approach of radiomics analysis of breast MRI for tumor decoding and phenotyping of breast cancer
Source: PLoS One. 2020 Jun 26;15(6):e0234871. doi: 10.1371/journal.pone.0234871 (PMC7319601; doi:10.1371/journal.pone.0234871)
Supplement: S3 File — This file contains a detailed description of the performed analysis. (DOCX) [file pone.0234871.s003.docx]

**Supplementary File 3**

**Analysis Pipeline**

**Flowchart**

184 patients identified from PACS

98 patients enrolled in study

Exclusion:

- patients with clip marker (N = 67)

- patients with BIRADS 4 (N = 3)

- patients with incomplete MRI (N = 16)

**Preprocessing**

Only patients with no missing examination were include in this study. Patients with missing outcome were removed from the corresponding sub-analysis. The corresponding scans and VOI segmentations were then downloaded from the PACS and anonymized. A simple threshold algorithm was applied on the VOI segmentation using 30% of the highest intensity inside the volume as threshold. The resulting shape was morphological closed. In case thresholding produced more than one lesion, only the largest lesion was retained. The resulting segmentation was then co-registered with all series.

Pyradiomics was then used to extract radiomics features from the segmentation and series. The parameters of pyradiomics were chosen based on previous experience: Normalization was enabled and a quantization binCount of 24 was used for all series.

**Feature Selection**

Resulting features with very small variation (<1e-5) were removed from the feature set. The features were scaled linearly to the range between 0 to 1. In case a feature value was N/A, it was replaced with 0. Shape features coming from segmentations other than the original segmentation were removed.

Five different feature selection methods were used to reduce the set of features: Randomized logistic regression, chi-square, f-score, t-score and mutual information. Implementations of the feature selection methods were taken from the sklearn.feature_selection module and used with default settings and without other changes. Randomized logistic regression is based on scikit 0.19.2 implementation RandomizedLogisticRegression with default parameters.

The feature selection methods were applied to all features at once. All feature selection except for randomized logistic regression produced a ranking and the top N features were selected. For randomized logistic regression, the coefficients of the trained model were used to rank the features.

**Analysis**

Three different machine learning algorithms were applied: Naive Bayes, random forests and logistic regression. Naive Bayes and logistic regression were not tuned. The 'lbfgs' optimizer was used for the logistic regression. For the random forest, a grid search using a stratified 2-fold cross-validation was used for tuning (strictly on the training fold, so a nested cross-validation scheme was adopted). As a full tuning of all parameters would be computationally too expensive, tuning parameters were chosen from previous experience as follows:

min_samples_split = [2]

n_estimators = [250]

max_features = ["auto"]

min_samples_leaf = [2,4,8]

max_depth = [5,10]

min_impurity_decrease = [0.005, 0.01]

**Validation**

Following the machine learning literature, a stratified 5-fold cross-validation (CV) with 25 repeats was used. In each repetition, the data set was split randomly into 5 folds. On 4 of these, the feature selection and the machine learning analysis was applied to yield a single model. This model was then used to obtain prediction on the left-out fold. After processing all folds, the predictions were pooled. This procedure was repeated 25 times. ROC analysis was then performed and mean AUC, Sensitivity and Specificity were computed. A two-sided DeLong test was employed to compare if the AUC of ROC curves is different to that of a constant predictor. Custom code was used, obtained from

https://github.com/yandexdataschool/roc_comparison.
