# Supplementary material for: A rapid volume of interest-based approach of radiomics analysis of breast MRI for tumor decoding and phenotyping of breast cancer
Source: PLoS One. 2020 Jun 26;15(6):e0234871. doi: 10.1371/journal.pone.0234871 (PMC7319601; doi:10.1371/journal.pone.0234871)

**Supplementary File 4**

**Violin plots**

These plots show the predicted scores (also called radiomics signature) for all patients, grouped by the corresponding outcome. The threshold was determined by the ROC analysis and is plotted as a horizontal line.
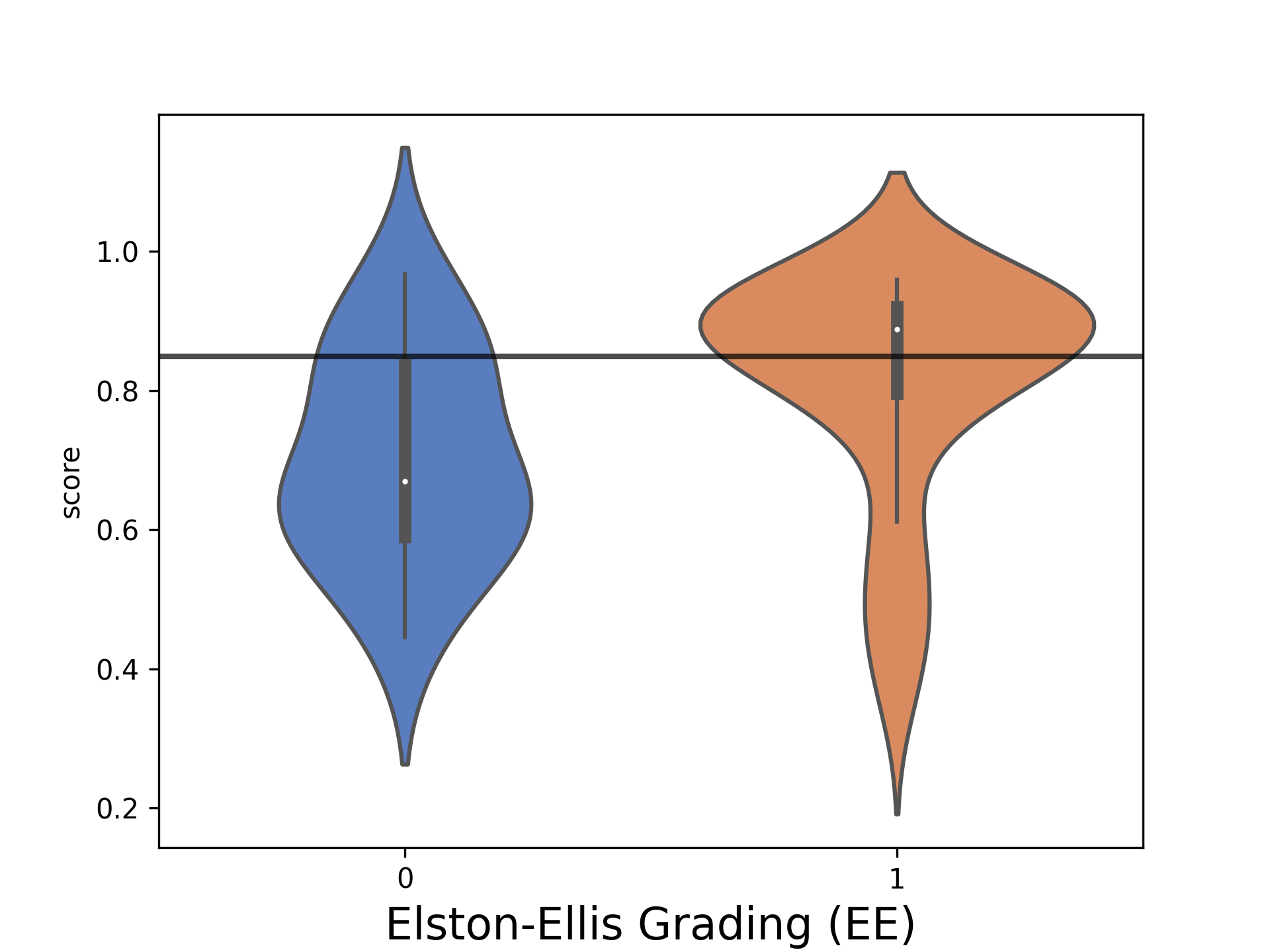

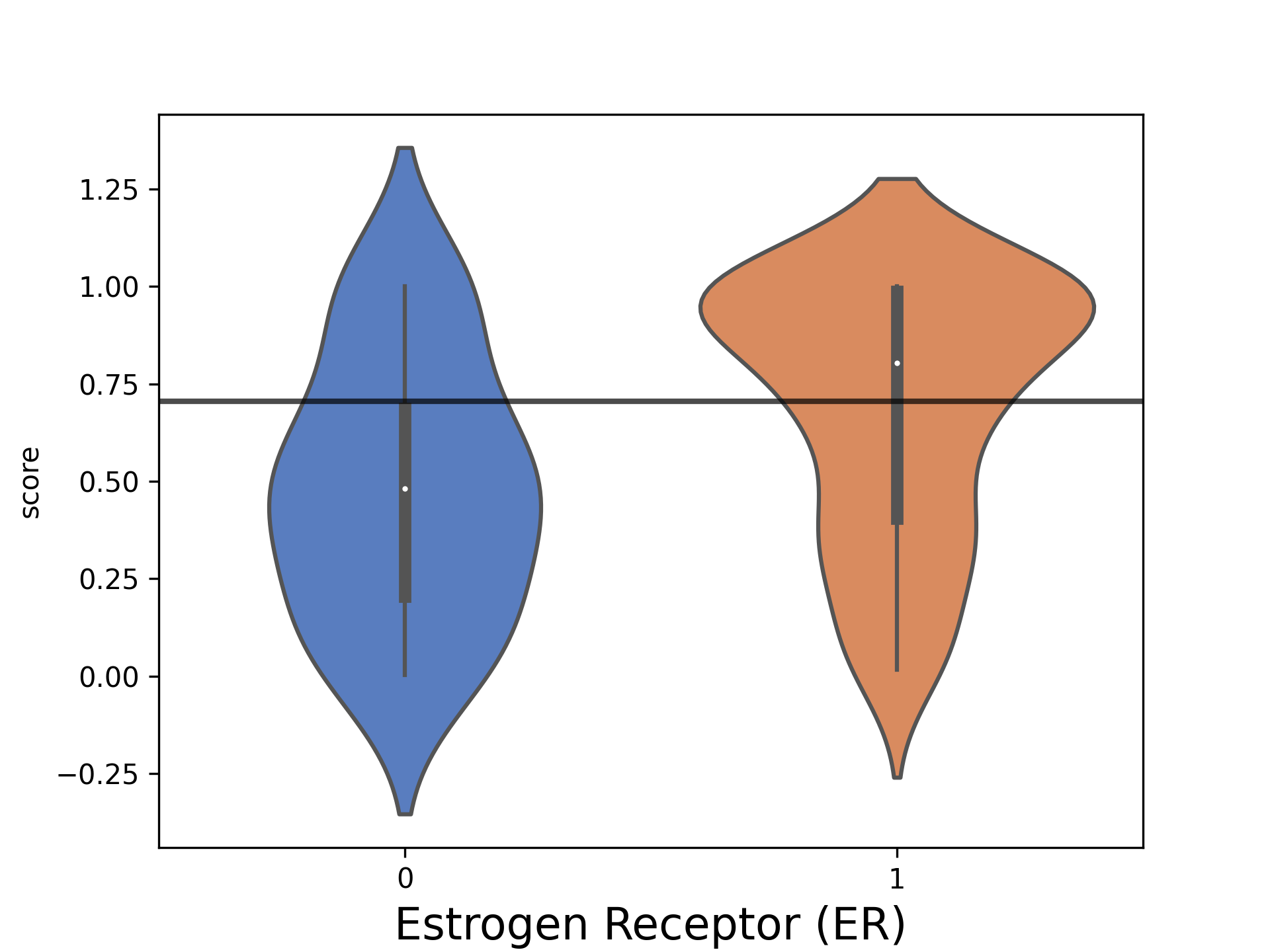

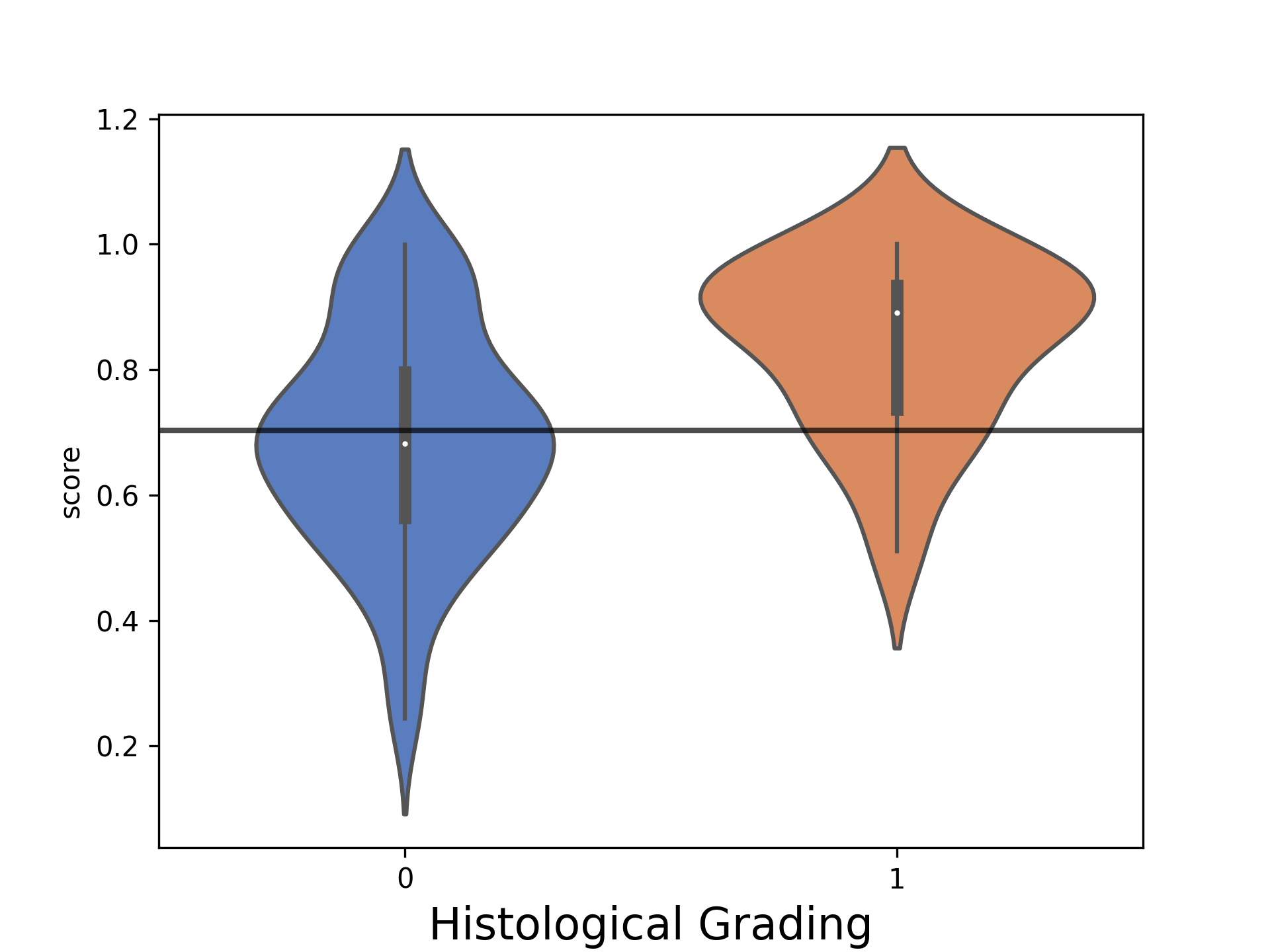

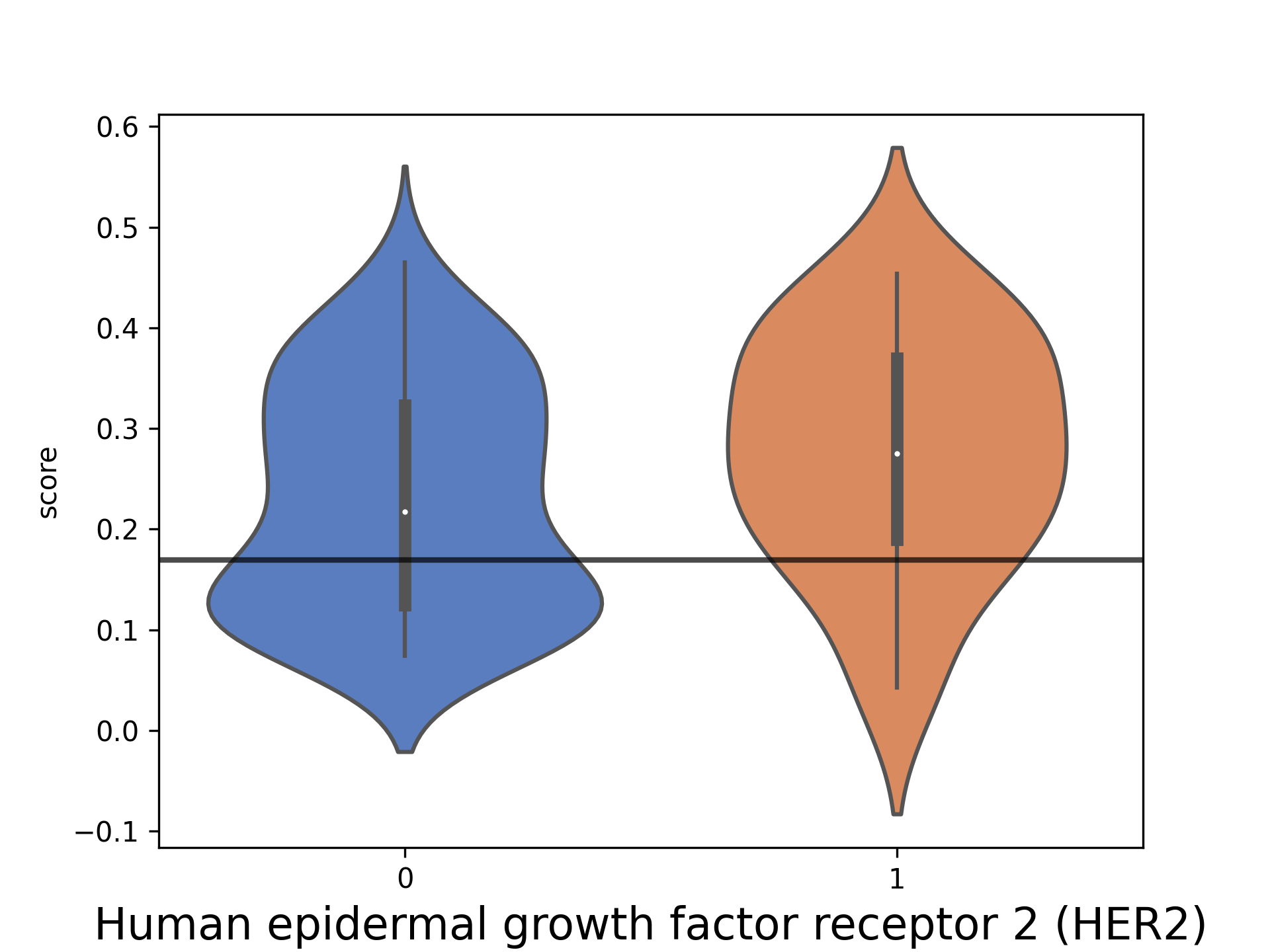

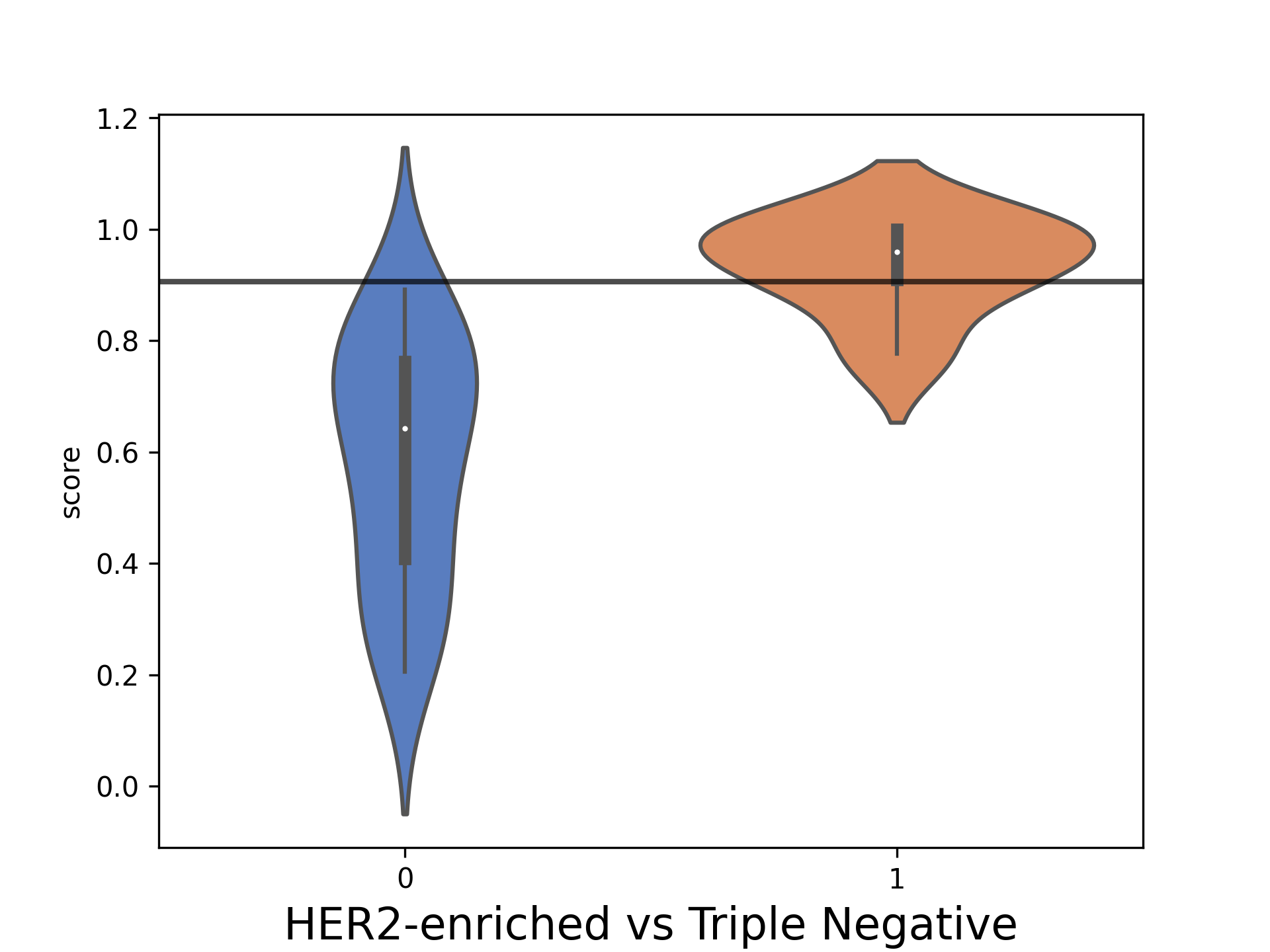

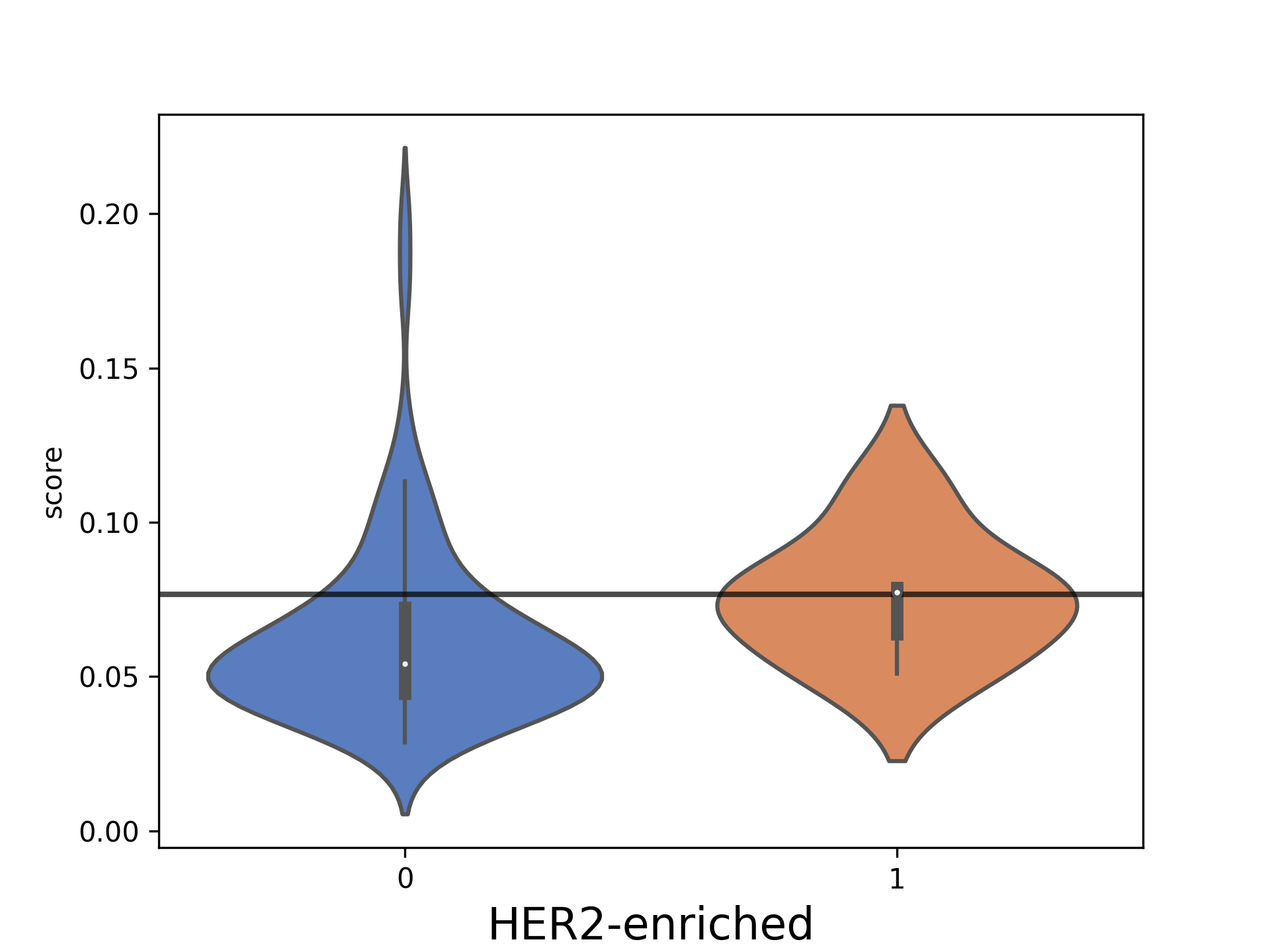

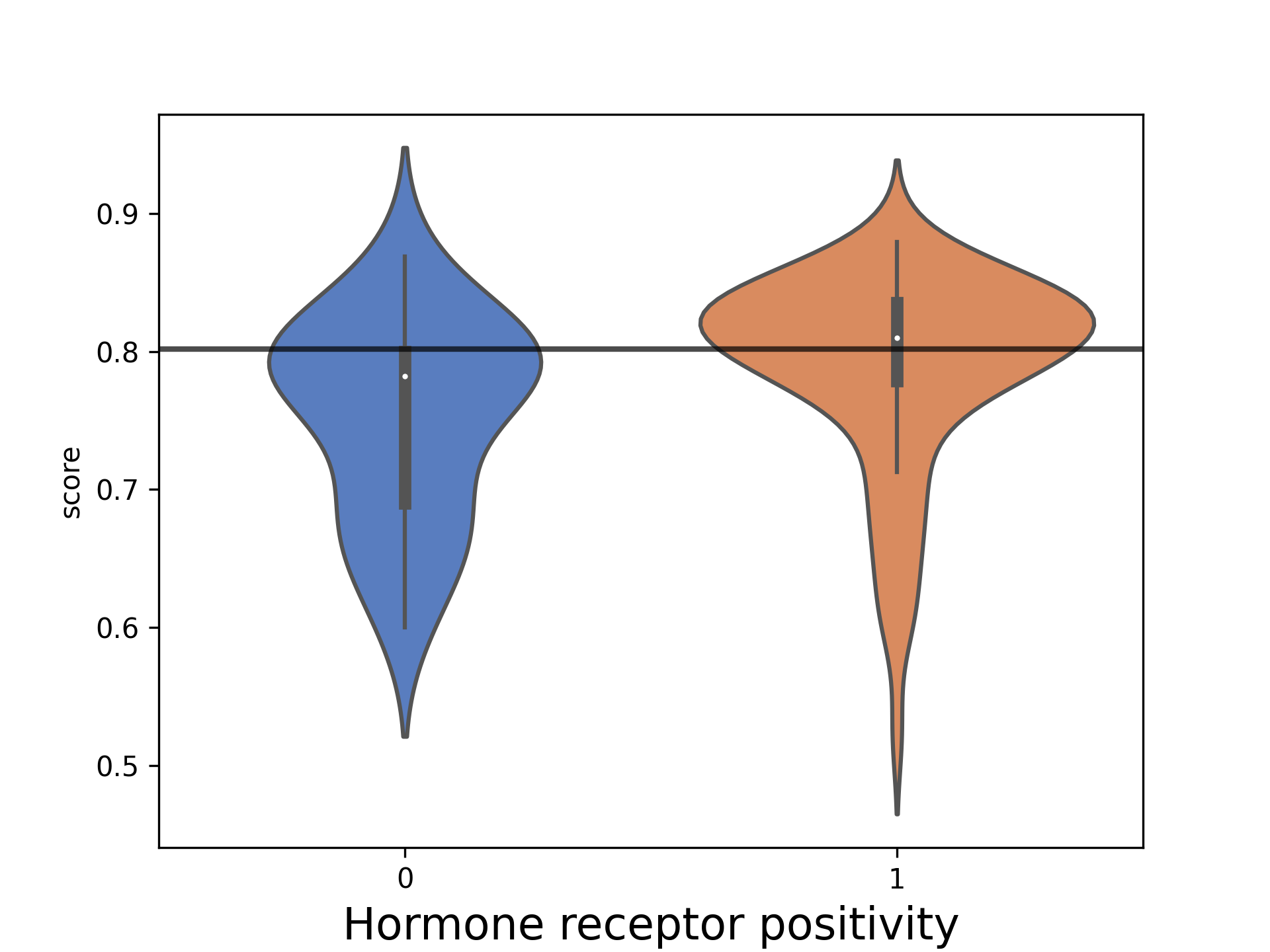

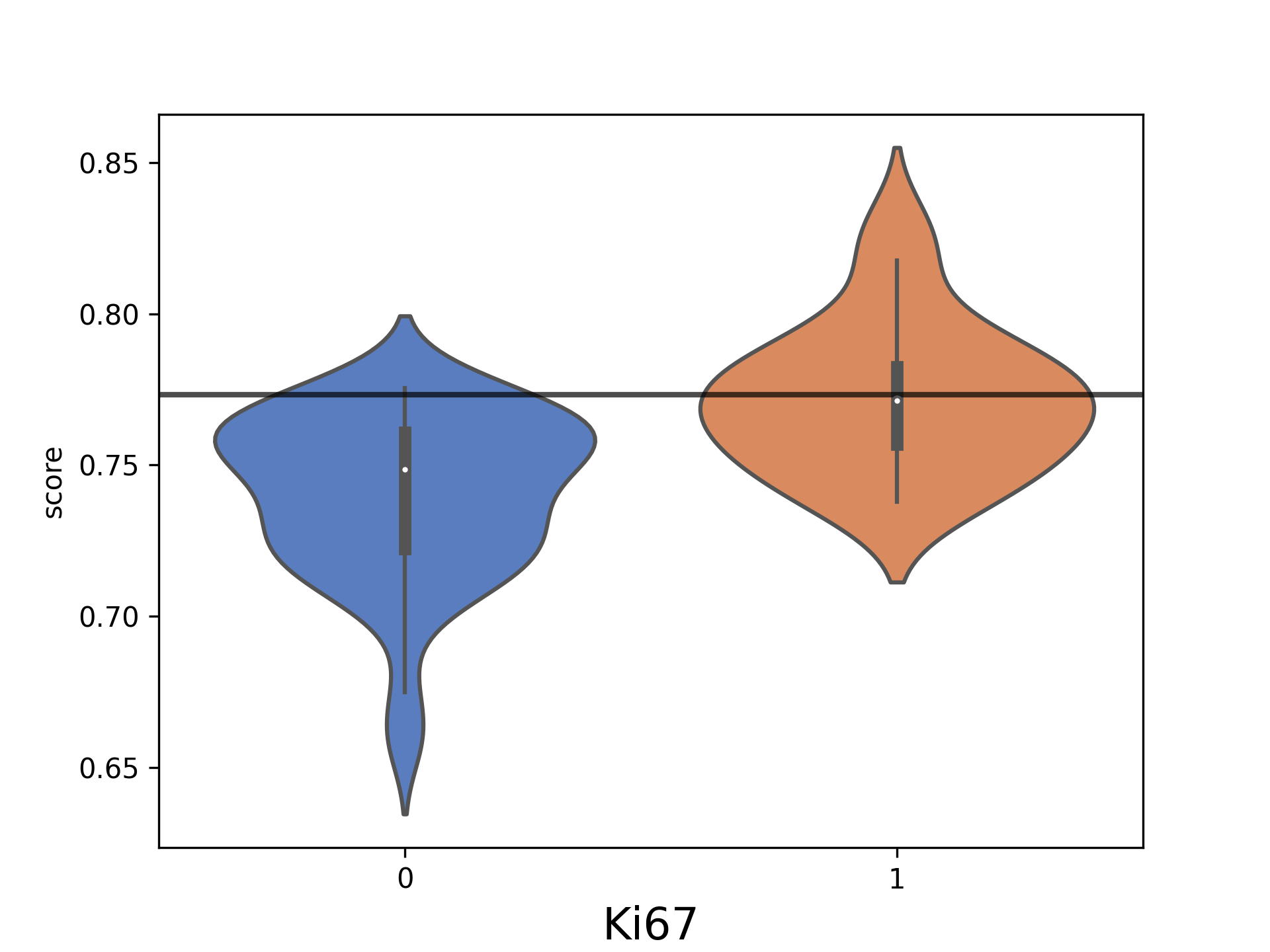

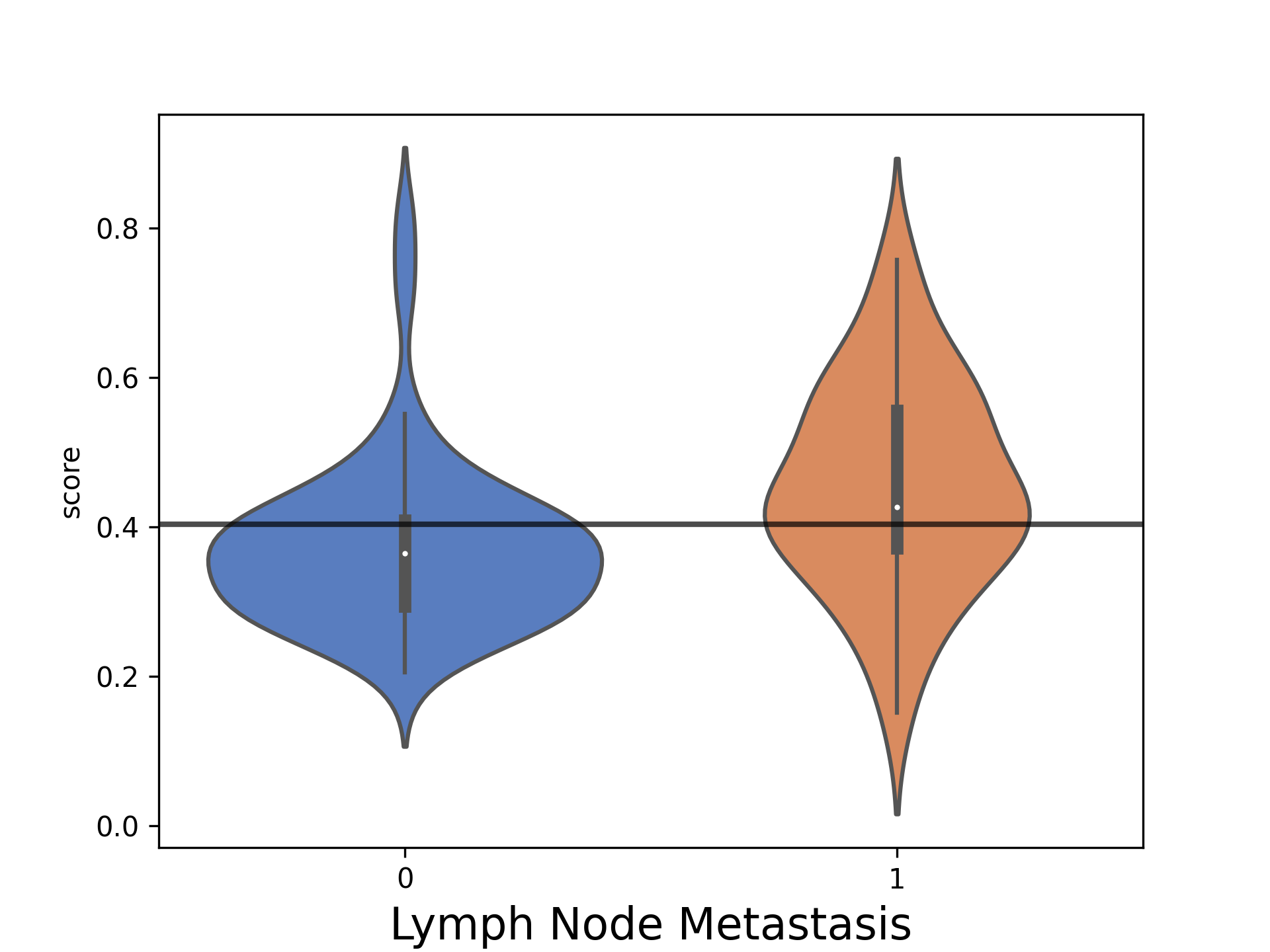

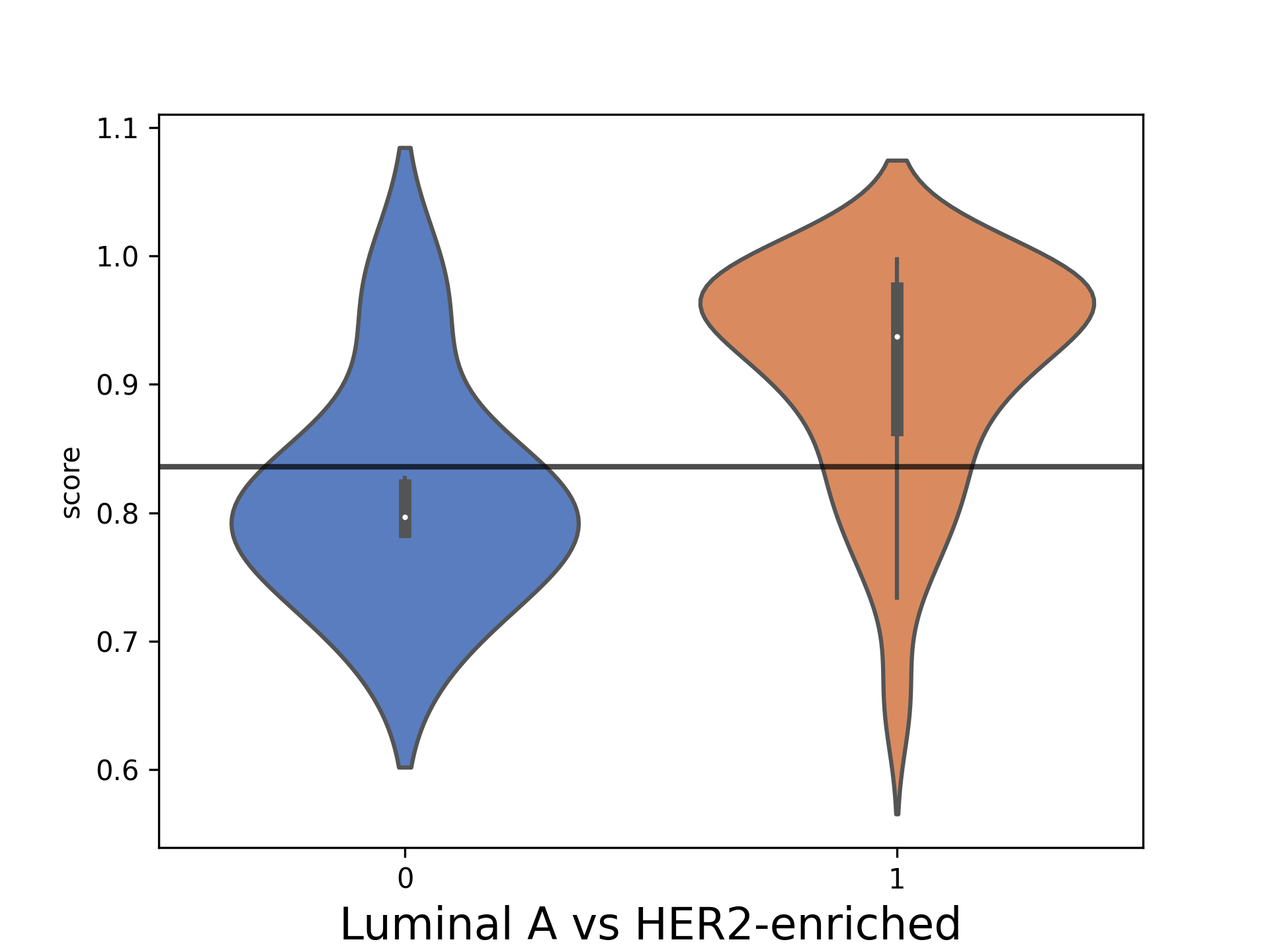

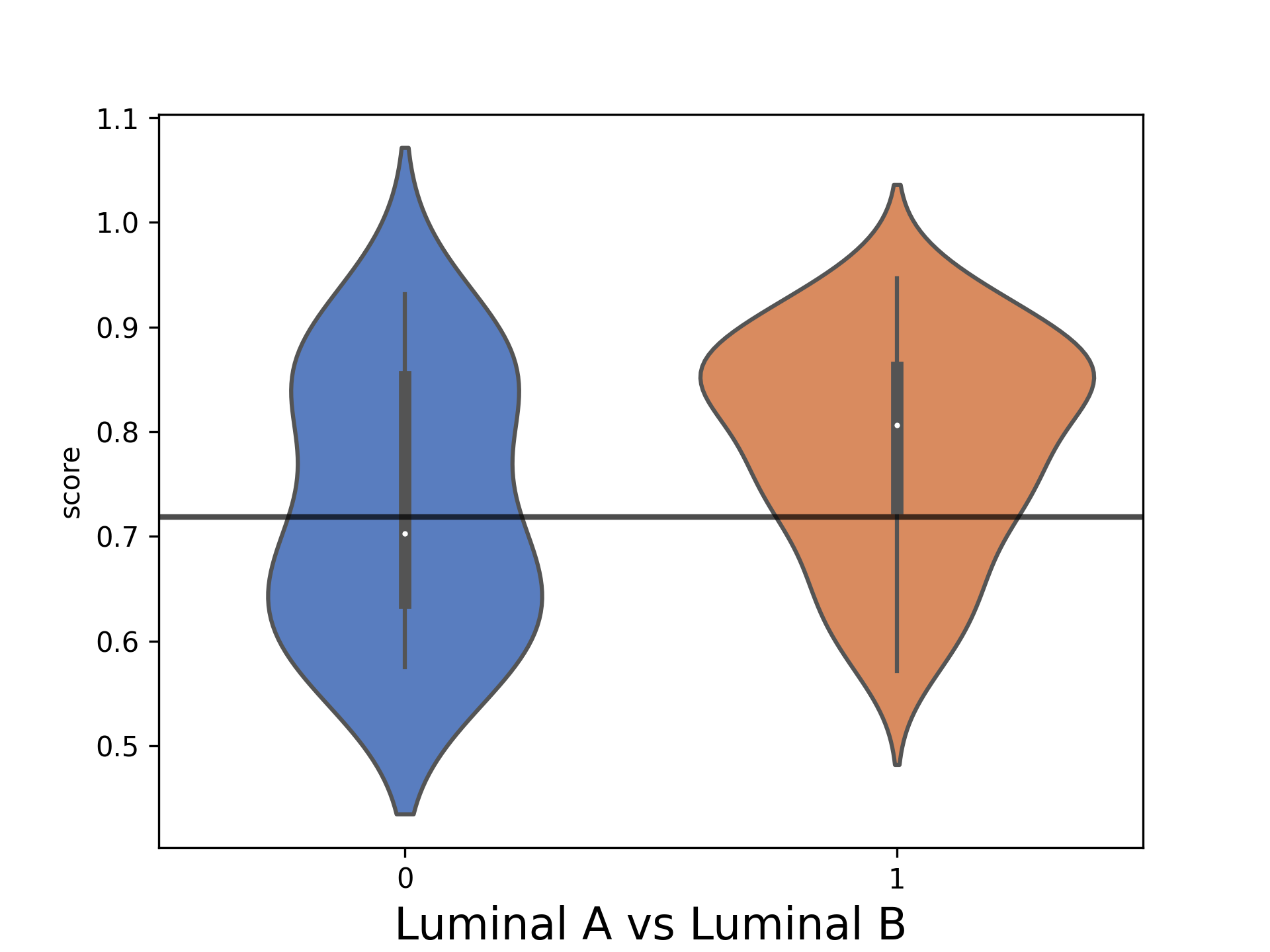

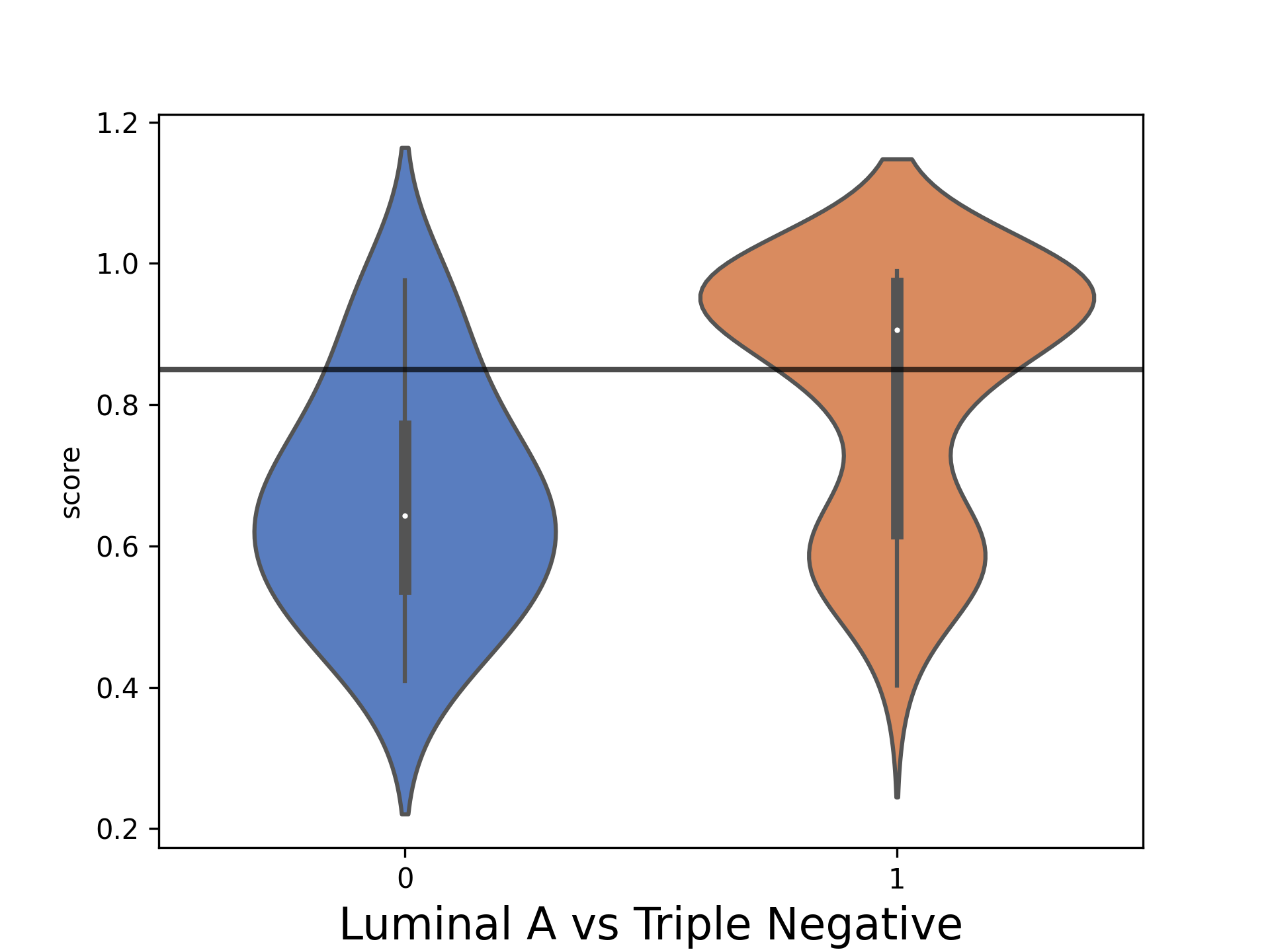

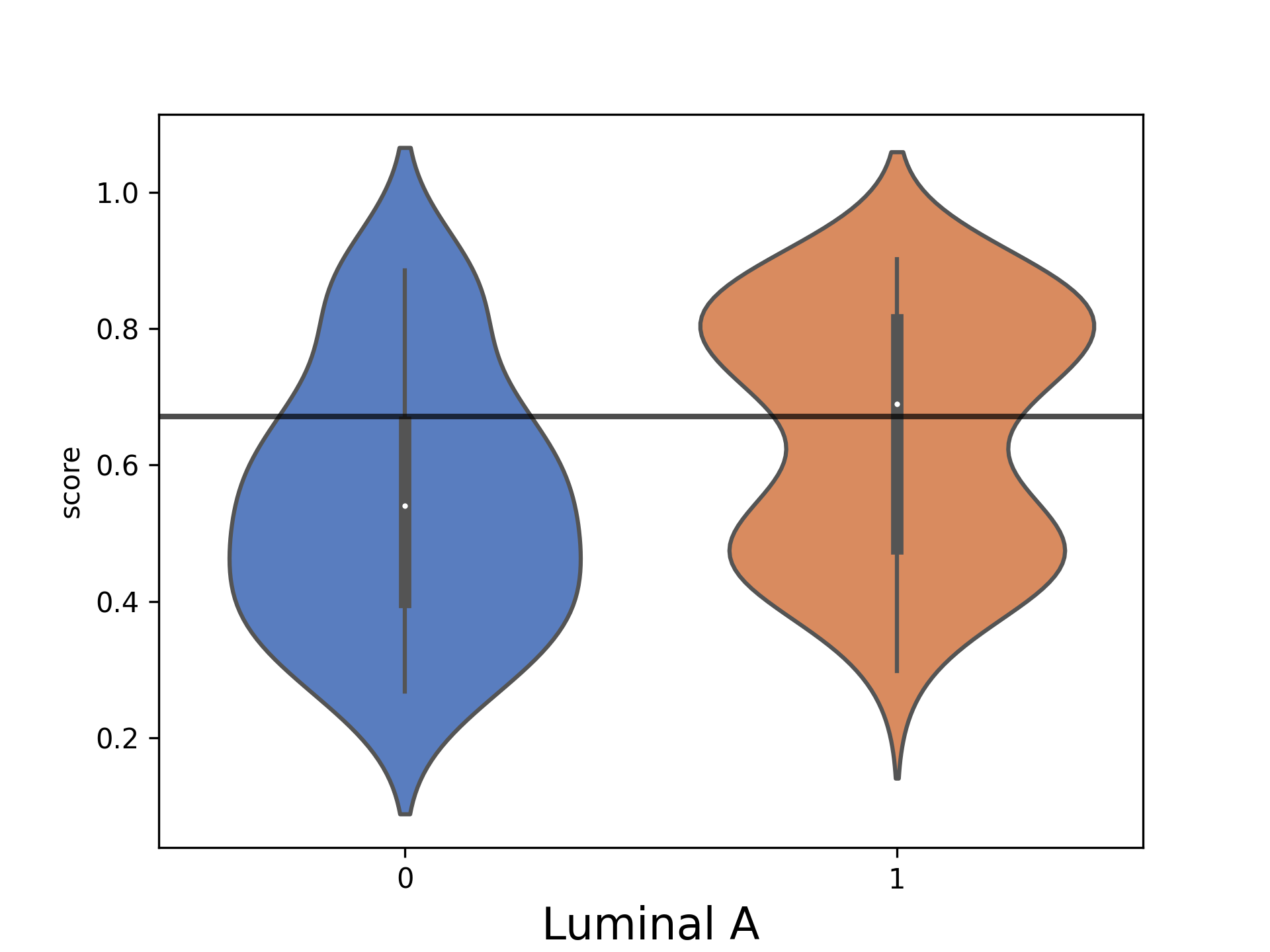

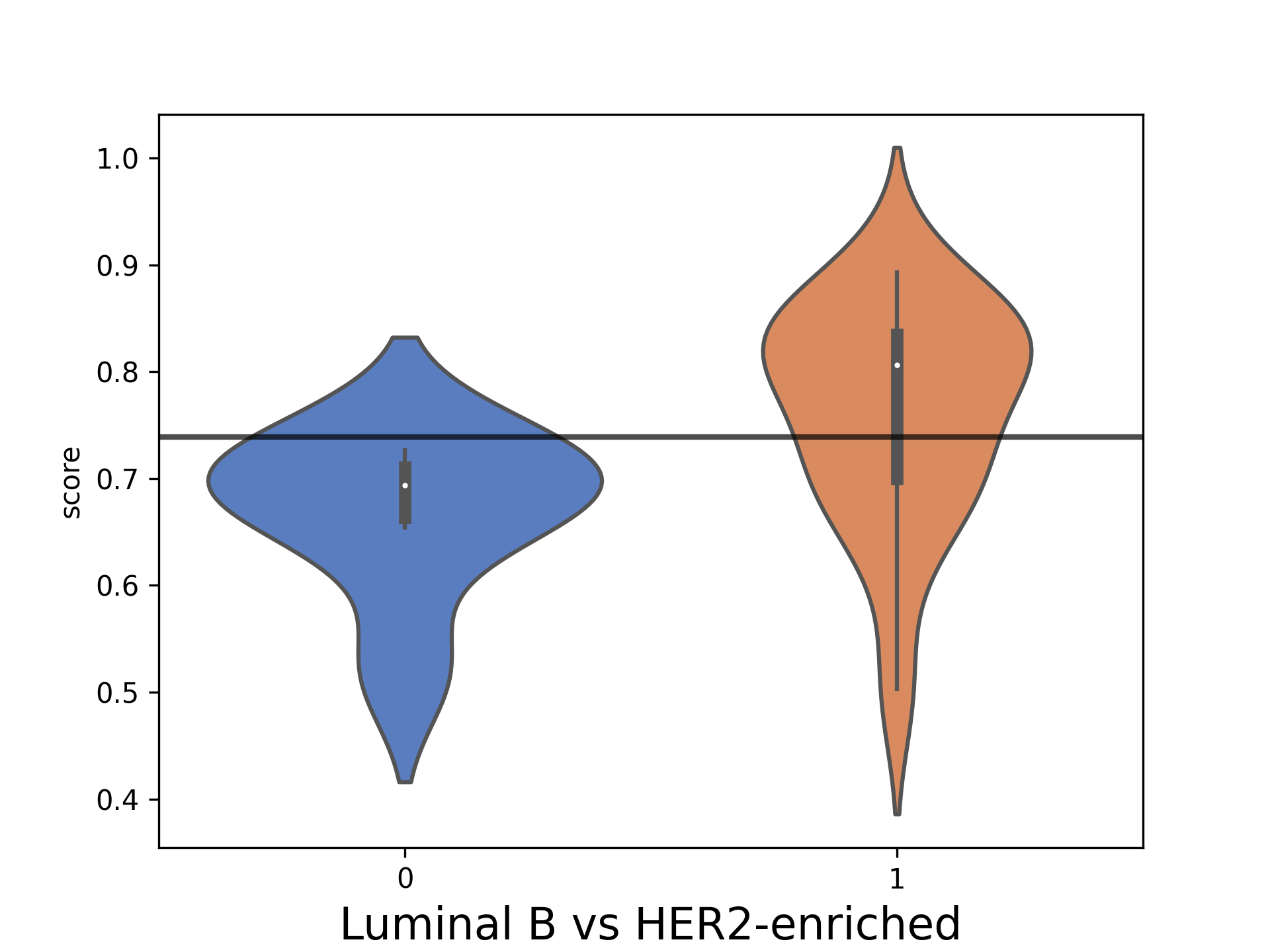

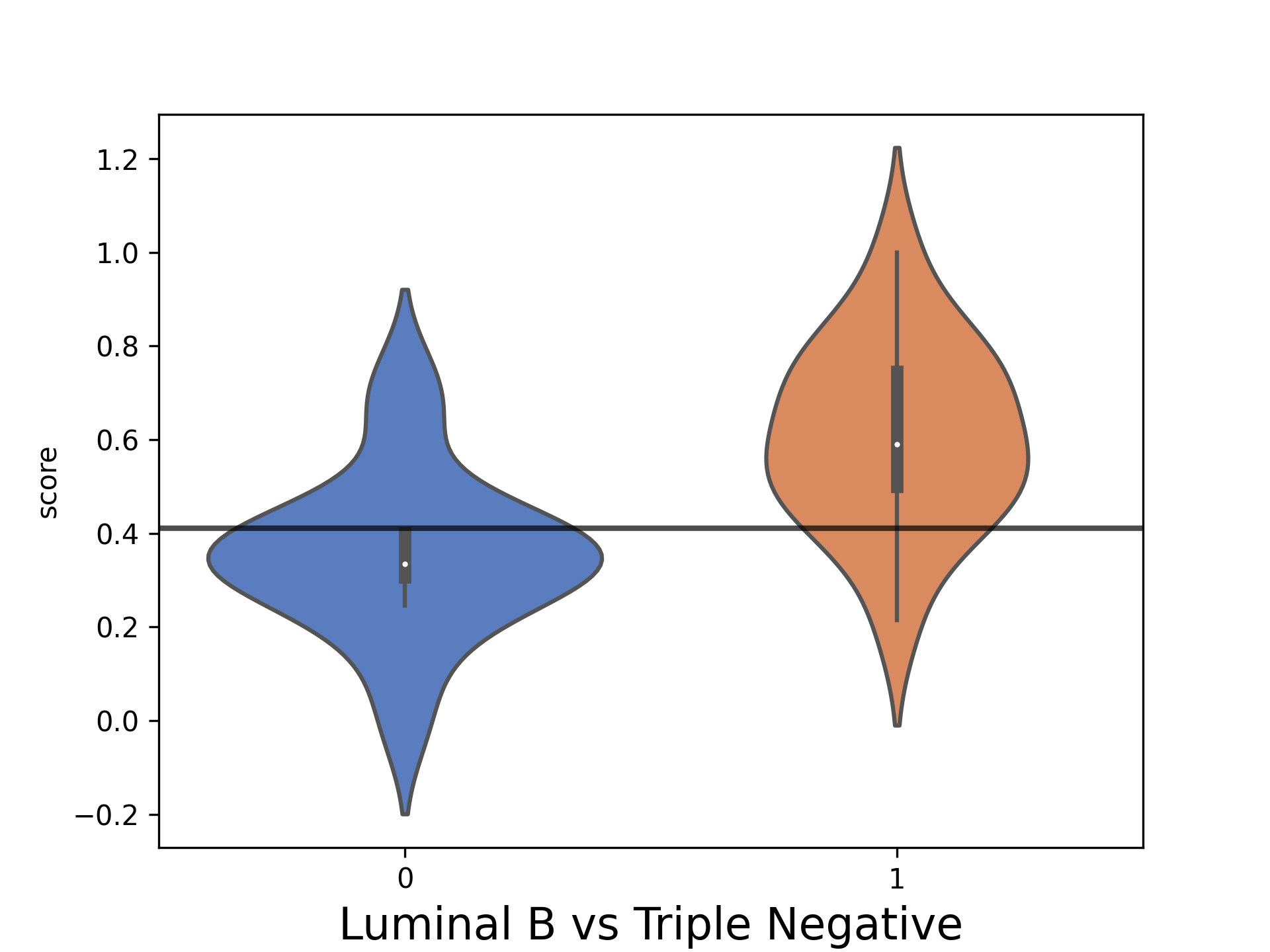

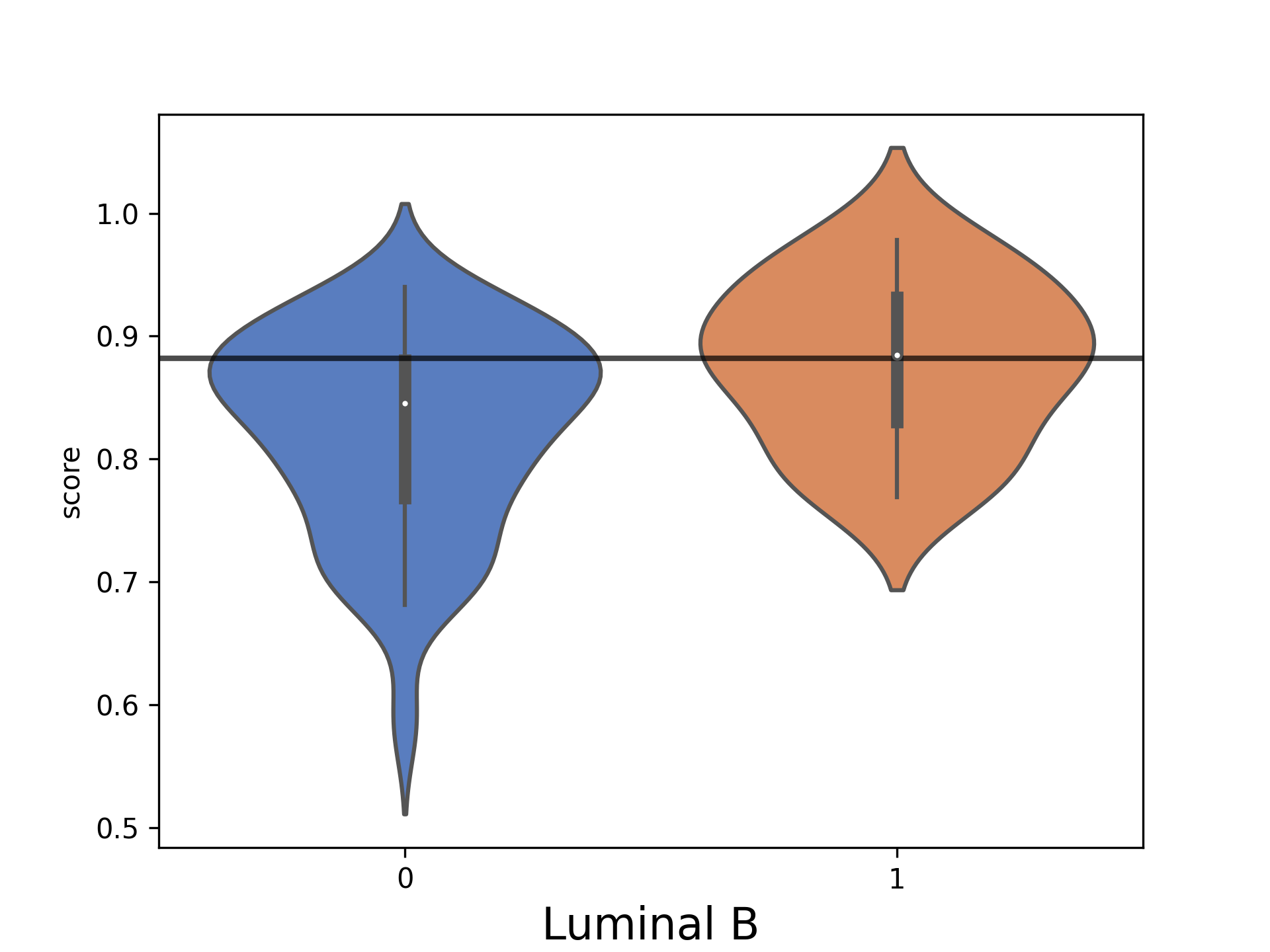

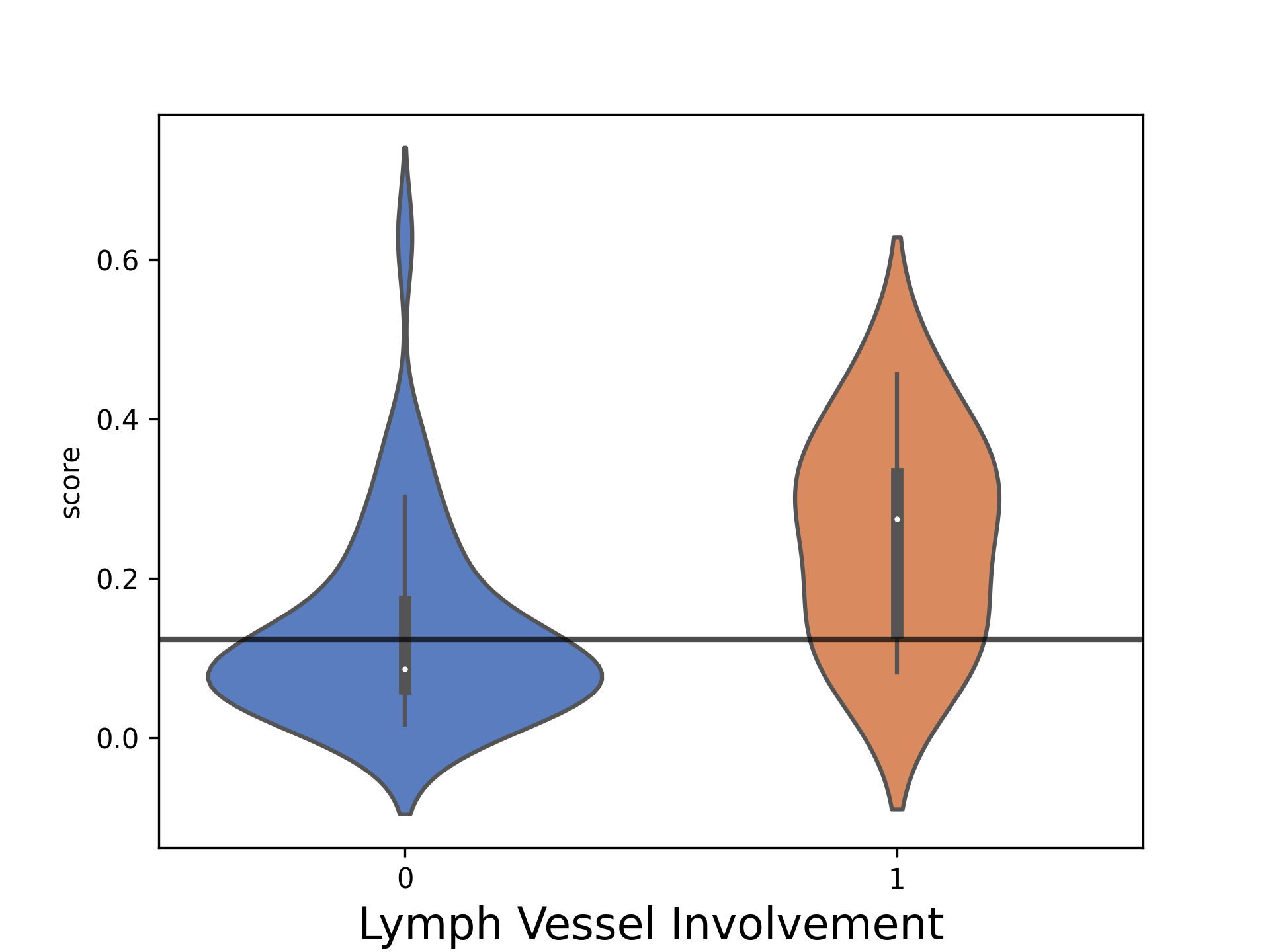

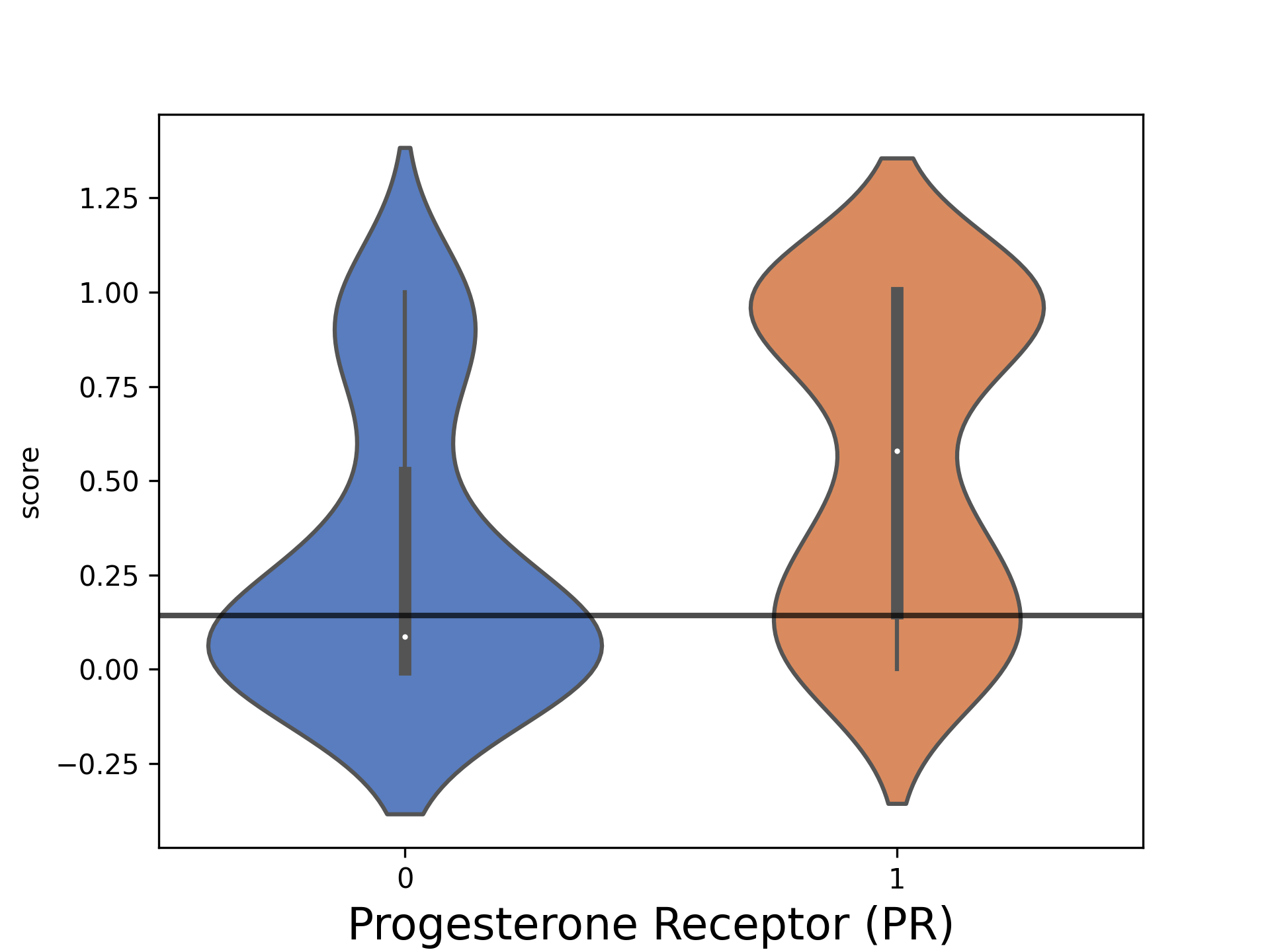

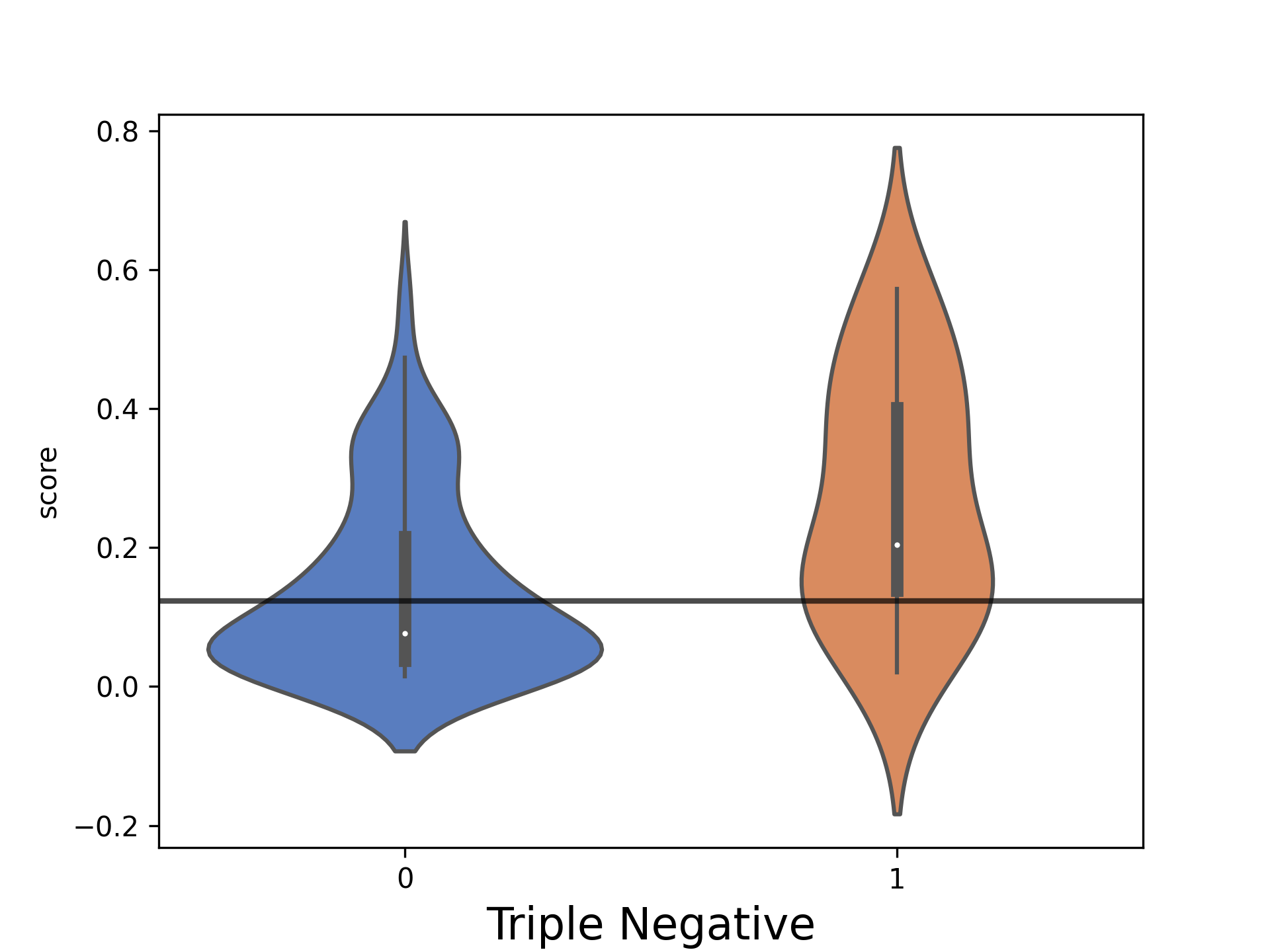

Supplement: S4 File — This file contains all violin plots for the prediction scores. (DOCX) [file pone.0234871.s004.docx]
