# Supplementary material for: A rapid volume of interest-based approach of radiomics analysis of breast MRI for tumor decoding and phenotyping of breast cancer
Source: PLoS One. 2020 Jun 26;15(6):e0234871. doi: 10.1371/journal.pone.0234871 (PMC7319601; doi:10.1371/journal.pone.0234871)
Supplement: S5 File — This file contains all ROC curves. (DOCX) [file pone.0234871.s005.docx]

**Supplementary File 5**

**ROC curves**
