# Supplementary material for: A rapid volume of interest-based approach of radiomics analysis of breast MRI for tumor decoding and phenotyping of breast cancer
Source: PLoS One. 2020 Jun 26;15(6):e0234871. doi: 10.1371/journal.pone.0234871 (PMC7319601; doi:10.1371/journal.pone.0234871)
Supplement: S6 File — This file contains a plot depicting the correlation of all extracted features with the outcomes. (DOCX) [file pone.0234871.s006.docx]

**Supplementary File 6**

**Correlations of extracted features with outcomes**

Pearson correlation coefficients between the extracted features and the outcomes are shown in this plot. Features were clustered by a hierarchical clustering algorithm.


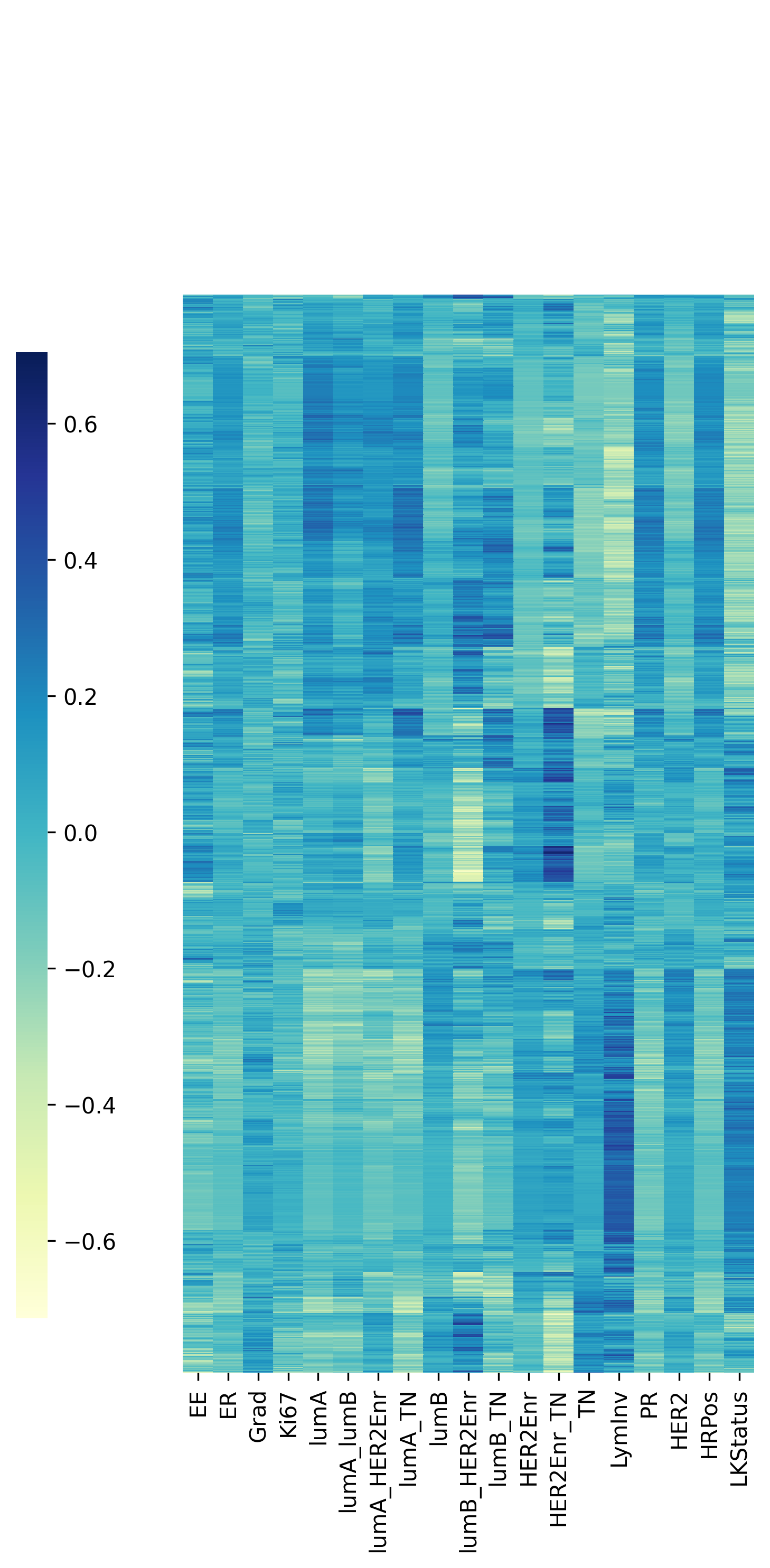


Legend:

EE = Elston-Ellis Grading (EE)

ER = Estrogen Receptor (ER)

PR = Progesterone Receptor (PR)

HRPos = Hormone receptor positivity

HER2Enr = HER2-enriched

TN = Triple Negative

lumA = Luminal A

lumB = Luminal B

lumA_lumB = Luminal A vs Luminal B

lumA_HER2Enr = Luminal A vs HER2-enriched

lumA_TN = Luminal A vs Triple Negative

lumB_HER2Enr = Luminal B vs HER2-enriched

lumB_TN = Luminal B vs Triple Negative

HER2Enr_TN = HER2-enriched vs Triple Negative

Ki67 = Ki67

HER2 = Human epidermal growth factor receptor 2 (HER2)

LymInv = Lymph Vessel Involvement

LKStatus = Lymph Node Metastasis

Grad = Histological Grading
